# Quantitative gene-level inference of tumor transcription from plasma epigenomes

**DOI:** 10.21203/rs.3.rs-11043657/v1

**Published:** 2026-10-05

**Authors:** Gunsagar S. Gulati, Damien Vasseur, Rashad Nawfal, Shahabeddin Sotudian, Karl Semaan, Marc Eid, Ji-Heui Seo, Shin Saito, Noa Phillips, John J. Canniff, Hunter Savignano, Surya B. Chhetri, Zhenjie Jin, Yukun Ou, Mohamed-Amine Bani, Renchin Wu, Maria-Anna Chrysovergi, Gwo-Shu Mary Lee, Rachel Trowbridge, Ilana B. Epstein, Gabriella Rickards, Paulo Roberto Da Silva Cordeiro, Ze Zhang, Razane El Hajj Chehade, Brady James, Christophe Massard, Antoine Italiano, Antoine Hollebecque, Jean-Charles Soria, Fabrice André, Cécile Badoual, Joaquim Bellmunt, Harshabad Singh, Andrew J. Aguirre, Brian M. Wolpin, Jacob Sands, Matthew G. Oser, Toni K. Choueiri, Sylvan C. Baca, Matthew L. Freedman

**Affiliations:** 1 Department of Medical Oncology, Dana-Farber Cancer Institute, Boston, MA, USA; 2 Center for Functional Cancer Epigenetics, Dana-Farber Cancer Institute, Boston, MA, USA; 3 Division of Population Sciences, Dana-Farber Cancer Institute, Boston, MA, USA; 4 Harvard Medical School, Boston, MA, USA; 5 Department of Medical Biology and Pathology, Gustave Roussy, Villejuif, France; 6 Paris Cité University, Paris, France; 7 Department of Pathology, Dana-Farber Cancer Institute, Boston, MA, USA; 8 Arthur and Linda Gelb Center for Translational Research, Dana-Farber Cancer Institute, Boston, MA, USA; 9 Department of Medical Oncology, Gustave Roussy, Villejuif, France; 10 Department of Therapeutic Innovation and Early Phase Trials, Gustave Roussy, Villejuif, France; 11 Paris-Saclay University, Department of Cancer Medicine, Gustave Roussy, Villejuif, France; 12 Lank Center for Genitourinary Oncology, Dana-Farber Cancer Institute, Boston, MA, USA; 13 Gastrointestinal Cancer Center, Dana-Farber Cancer Institute, Boston, MA, USA; 14 Lowe Center for Thoracic Oncology, Dana-Farber Cancer Institute, Boston, MA, USA; 15 These authors contributed equally.; 16 Senior authors.; 17 Lead contact.

**Keywords:** cell-free chromatin, cfChIP-seq, plasma epigenomics, gene expression inference, chromatin-based transcriptional profiling, liquid biopsy, histone modifications, machine learning, precision oncology, antibody-drug conjugates, pancreatic cancer, small-cell lung cancer, bladder cancer

## Abstract

Tumor phenotype and therapeutic vulnerability are shaped by dynamic transcriptional states that are not captured by genetic profiling alone and are difficult to monitor longitudinally. Existing plasma-based approaches have largely been developed using samples with high circulating tumor DNA fractions, evaluated across expression quantiles rather than individual genes, and rarely benchmarked head-to-head. Using timepoint-matched plasma epigenomes and tumor transcriptomes, we identify biologically grounded relationships between circulating chromatin, fragmentation, and transcription. H3K4me3 and H3K36me3 encode distinct positional information reflecting promoter activity and transcriptional elongation, while gene-body fragment periodicity predicts expression. We integrate these relationships into APEX, a machine-learning framework for genome-wide single-gene expression inference. Across 559 plasma samples spanning 24 cancer types and clinically relevant tumor fractions, APEX outperforms existing approaches for individual-gene inference, resolves pancreatic cancer transcriptional subtypes, tracks therapeutic targets in small-cell lung cancer, and identifies NECTIN4 expression as a biomarker of response to enfortumab vedotin in urothelial cancer.

## INTRODUCTION

Many clinically actionable features of cancer, including lineage state, therapeutic target expression, and non-genetic mechanisms of drug resistance, are defined by transcriptional rather than genomic alterations. As targeted biologics against variably expressed cell-surface proteins expand and non-genetic mechanisms of therapy resistance become increasingly recognized, there is a growing need to measure tumor gene expression dynamically and noninvasively^1–4^. However, assessment of tumor transcriptional state still relies largely on tissue biopsies, which are limited by sampling bias, spatial heterogeneity, and the difficulty of longitudinal tumor sampling.

Liquid biopsy offers a noninvasive alternative, but most cell-free nucleic-acid assays focus on genomic alterations, with limited approaches available to infer tumor transcriptional activity from blood^5^. Although cell-free RNA (cfRNA) can provide information about tissue and cell-type composition, its analysis is complicated by RNA instability, heterogeneous cellular origins, and technical variability^6,7^, motivating complementary strategies that infer transcription from the more stable chromatin features retained by cell-free DNA (cfDNA).

cfDNA circulates as protein-protected fragments that retain information about the chromatin and regulatory states of their cells of origin^8^. Fragment length, positioning, and sequence composition have each been associated with gene expression^9–15^, while immunoprecipitation of histone modifications and DNA methylation can further enrich regulatory information from circulating chromatin^8,16–21^. However, how these distinct and potentially complementary signals quantitatively relate to transcription in the tissue of origin remains incompletely understood. Defining these relationships could enable more accurate reconstruction of tumor gene expression from plasma.

Existing cell-free epigenomic approaches have largely focused on classification of tissues, cancer types, or broad transcriptional programs rather than quantitative inference of individual tumor genes (**Table S1**). Performance has commonly been evaluated across gene-expression bins or aggregate transcriptional programs, leaving the accuracy of individual-gene predictions incompletely characterized. This limits applications requiring gene-level precision, including measurement of therapeutic targets and single-gene biomarkers, while sensitivity at clinically relevant plasma tumor fractions, like the median ~3% observed in metastatic solid cancers^22^, remains an additional challenge. Thus, realizing the potential of plasma epigenomics requires both a better understanding of how circulating chromatin encodes transcription and methods that leverage these signals for accurate individual-gene inference at low tumor fractions.

Here, we demonstrate that histone modifications and DNA fragmentation in circulating chromatin provide complementary, position-dependent signals of tumor gene expression across regulatory regions. We develop APEX (Associating Plasma Epigenomics with eXpression), a machine-learning framework that integrates these features to infer genome-wide tumor gene expression at single-gene resolution from plasma. Trained using pan-cancer cohorts with matched cfChIP-seq and tumor RNA-seq, APEX outperforms existing chromatin- and fragmentomics-based approaches in head-to-head comparisons and retains performance across clinically relevant tumor fractions. Across 559 plasma samples comprising 1,274 epigenomic profiles from 24 cancer types, APEX resolves clinically relevant pancreatic cancer transcriptional states, longitudinally tracks therapeutic cell-surface targets during treatment in small-cell lung cancer, and identifies plasma-inferred expression biomarkers associated with treatment response in urothelial cancer.

## RESULTS

### Cell-free chromatin features linked to tumor gene expression

We first sought to determine whether the epigenomic organization and fragmentation of circulating chromatin could provide quantitative information about tumor gene expression. We analyzed 15 patients across seven cancer types enrolled in the MOSCATO–1 clinical trial^23^, each with tumor RNA-seq and plasma collected within 24 hours of biopsy (Figure 1A; **Table S2**). Plasma tumor fractions spanned <3% to 42% (median, 14%) by ichorCNA^24^, enabling evaluation across a broad range of circulating tumor DNA abundance.

To capture complementary regulatory information from circulating chromatin, we profiled H3K4me3 and H3K27ac, which mark active promoters and enhancers, together with the gene body-associated mark H3K36me3 using serial cell-free chromatin immunoprecipitation and sequencing (cfChIP-seq).^17^ We selected H3K36me3 after benchmarking six histone marks for recovery from plasma (**Figures S1 and S2; Table S3**). Although several marks were strongly associated with transcription in bulk tissues and cell lines, H3K36me3 provided broader genomic occupancy, more robust gene-body enrichment in plasma, and stronger lineage- and expression-associated signals (**Figures S1 and S2**).

We next systematically mapped the relationship between circulating chromatin and tumor transcription across 17,950 protein-coding genes. For each gene, we defined promoter (±3 kb around the transcription start site [TSS]), gene body (TSS to transcription termination site [TTS]), and, where annotated, intra- and intergenic enhancer regions using GeneHancer associations^25^ (Figure 1B; **Table S4**). These regions were subdivided into bins to resolve spatial variation in chromatin signal. From plasma cfChIP-seq and matched low-pass whole-genome sequencing (LPWGS), we extracted 3,516 candidate features capturing histone coverage, fragment-length distributions, GC content, and 4-mer end-motif composition (Figures 1B and 1C; **Table S5**). We then quantified the association between each circulating chromatin feature and matched tumor RNA-seq expression across genes (Figure 1C).

This unbiased screen revealed extensive and spatially organized relationships between circulating chromatin and tumor transcription (Figure 1D). H3K4me3 coverage was most strongly associated with expression immediately downstream of the TSS, with maximal associations within the proximal 0–20% of the gene body (Figures 1D and 1E). By contrast, H3K36me3 progressively increased across transcribed regions and was most strongly associated with expression toward the 3′ gene body, consistent with its known coupling to transcriptional elongation (Figures 1D and 1E)^26^. These relationships were evident genome-wide, as genes with progressively higher tumor expression showed corresponding enrichment of promoter-proximal H3K4me3 and gene-body H3K36me3 in matched plasma (Figure 1F). H3K27ac and enhancer-associated signals were comparatively modest, while coverage from unenriched LPWGS showed weak associations with expression, indicating that the observed relationships were not explained by total cfDNA abundance alone (Figures 1D and 1E). Fragment-length entropy, end-motif diversity, and promoter-associated GC content also carried transcription-associated information, whereas mean fragment length, fragment-length variance, and individual 4-mer sequences contributed comparatively little (**Figure S3**).

Unexpectedly, fragment size within transcription-associated circulating chromatin provided additional information about tumor expression. Within H3K36me3-enriched gene bodies, highly expressed genes were associated with enrichment of subnucleosomal fragments (80–120 bp) and di- and tri-nucleosomal fragments (280–320 bp and 400–440 bp), together with depletion of mononucleosomal fragments (160–200 bp) (Figures 1G and 1H; **Figure S4**). The same oscillatory relationship between fragment size and gene expression was observed in deeply sequenced unenriched plasma cfDNA (120× and 138×) from two additional cancer patients with matched tumor RNA-seq (Figure 1H)^9,11^.

We hypothesized that these fragment-expression relationships reflect differences in nucleosome organization between transcriptionally active and inactive chromatin. To test this directly, we compared H3K36me3-associated fragments generated from LNCaP nuclei by micrococcal nuclease (MNase) digestion, which preferentially cleaves accessible linker DNA while preserving nucleosome-associated fragments, with fragments generated by random mechanical shearing. MNase-derived fragments recapitulated the periodic relationship between fragment size and gene expression observed in plasma, whereas the relationship was largely removed following sonication (Figure 1H). These findings support a model in which transcription-associated nucleosome organization produces characteristic fragment-length distributions that are retained in circulating chromatin (Figure 1I).

Collectively, these observations suggested that complementary features of circulating chromatin could be integrated to infer gene expression directly from plasma.

### Development of APEX

We developed APEX (Associating Plasma Epigenomics with eXpression), a machine-learning framework for inferring tumor gene expression from circulating chromatin (Figure 2A; Methods). Rather than fitting separate models for individual genes, APEX learns a shared relationship between local plasma chromatin and fragmentomic features and tumor transcription across protein-coding genes. We evaluated nine epigenomic feature classes derived from H3K4me3, H3K27ac, and H3K36me3 cfChIP-seq measurements across binned promoters, enhancers, and gene body regions (Figures 2A and **S5A**; **Table S6**). After benchmarking multiple regression approaches, we selected XGBoost based on cross-validated prediction accuracy and optimized feature normalization, genomic region definitions, and model hyperparameters using patient-level cross-validation against matched tumor RNA-seq (**Figures S5B–S5O**).

Because many genes use alternative promoters, we asked whether sample-specific TSS selection could improve inference of promoter activity. To do this, we assigned each gene a sample-specific TSS corresponding to the annotated TSS with maximal local H3K4me3 signal. This significantly improved the association between promoter coverage and matched tumor expression, particularly for genes with distal alternative promoters (**Figure S6**). However, the benefit of sample-specific promoter selection diminished at lower tumor fractions, consistent with increasing contribution from background non-tumor promoter signal (**Figure S7**).

### Interpretation of APEX

We next evaluated the contributions of individual histone marks, genomic regions, and feature classes using in silico ablation and permutation analyses with patient-level leave-one-out cross-validation. H3K4me3 and H3K36me3 each contributed substantially to predictive performance, whereas H3K27ac provided little additional benefit in either the original training cohort or the independent 34-patient validation cohort described below (**Figures S8A and S9**). Promoter and gene-body features were more informative than enhancer-derived features, and DNA methylation provided only a modest incremental improvement (**Figures S8B and S8C**). Feature-group ablation further showed that no single feature class recapitulated the performance of the integrated model (**Figure S8D**). Consistent with this, applying the same feature architecture to deeply sequenced cell-free DNA whole-genome sequencing without immunoprecipitation yielded lower prediction accuracy than cfChIP-seq, indicating that sequencing depth and fragmentomic information alone do not account for APEX performance (**Figure S10**).

Permutation-based feature-importance analysis identified H3K4me3 coverage within the proximal gene body, H3K36me3 fragment frequencies of 160–200 bp, and maximum H3K36me3 gene-body coverage among the strongest predictors (Figure 2B; **Table S6**). Based on these collective analyses, we refined APEX to only H3K4me3- and H3K36me3-derived promoter and gene-body features. This focused model retained the accuracy of the full feature set while reducing runtime and simplifying assay implementation (**Figures S8E–S8H**).

Finally, to assess the reproducibility of the learned feature architecture, we trained APEX de novo in an independent cohort of matched plasma–tumor pairs. Despite independent training, 9 of the 10 highest-ranked features were shared between models, and feature-importance rankings were strongly concordant across all 1,532 features (Spearman ρ = 0.84; **Figure S11**).

APEX achieved a median single-gene Spearman correlation of 0.80 under patient-level leave-one-out cross-validation. Performance approached the null distribution after permuting tumor expression labels, remained stable after substantial downsampling, and was robust to moderate noise in tumor RNA-seq measurements (**Figures S12A–S12C**). Moreover, leave-one-cancer-type-out performance was comparable to leave-one-patient-out performance, supporting generalization to cancer types not represented during training (**Figure S12D**).

### Performance of APEX

To independently validate APEX, we applied the fixed model developed in the 15-patient training cohort to matched plasma and tumor samples from 34 additional patients spanning 18 cancer types, including 11 not represented during training, with tumor fractions comparable to those in the training cohort (Figures 2C and **S12E**; **Table S7**). The model consistently demonstrated high correlations with matched tumor RNA-seq across cancer types, achieving comparably high performance compared to the training cohort and outperforming individual histone mark coverage (Figures 2D–2F and **S12F**). Performance was not restricted to white blood cell (WBC) background, defined as genes expressed at higher levels in whole blood compared to other tissues based on public RNA-seq data, and remained high for non-WBC genes, with correlation performance being superior to individual histone marks across tumor fractions (**Figures S12G and S12H**).

We next examined whether APEX prediction accuracy depended systematically on gene characteristics. In the independent validation cohort, prediction error was modestly affected by expression level, tissue specificity, WBC association, gene length, and promoter CpG-island status. Multivariable analysis indicated that no single characteristic accounted for performance across genes (**Figure S13**). We then asked whether models trained separately within defined gene categories could improve prediction. Category-specific models stratified by expression level, gene length, housekeeping status, transcription-factor identity, or tissue specificity performed similarly to the global APEX model, suggesting that both approaches captured largely overlapping predictive information and supporting the use of a single cross-gene model in the current dataset (**Figure S14**).

To directly evaluate the effect of tumor fraction on APEX’s ability to detect cancer-specific signal, we serially diluted tumor-derived plasma cell-free chromatin from three separate patients with small-cell lung cancer with equimolar non-cancer donor plasma (Methods; Figure 2G). We then quantified the limit of detection for SCLC gene expression using a published approach^14^. Compared to individual histone marks, APEX detected expression of a SCLC signature at a lower tumor fraction (0.1% vs 1.2% and 3.8%; Figure 2H) and detected more individual SCLC genes, such as *CHGA* and *DLL3*^27,28^ (78% vs 30% and 5%; Figures 2I and **S15**), at a statistically significant threshold.

We further tested whether APEX could retain more granular tumor-specific information as tumor fraction decreased. Using plasma from two patients with matched biopsies from distinct anatomical sites, we defined primary-lung- and lymph-node-specific expression signatures from the corresponding tumor RNA-seq profiles. Across serial in silico dilution, APEX preferentially recovered the transcriptional program of the biopsy site matched to each plasma sample, while enrichment of the unmatched site-specific program remained minimal (**Figure S16**). These findings indicate that APEX can preserve patient- and site-specific transcriptional differences even as tumor-derived signal is progressively diluted.

### Benchmarking of APEX

To benchmark APEX against existing approaches for inferring gene expression from plasma, we selected five samples from the independent validation cohort with sufficient cell-free DNA (20–100 ng) for matched cfChIP-seq and deep whole-genome sequencing (mean coverage, 103×; **Table S9**). We implemented published cfChIP-seq- and fragmentomics-based approaches on the same samples and compared inferred expression directly with matched tumor RNA-seq. APEX achieved a median single-gene Spearman correlation of 0.82, compared with 0.61 for the next-best performing method^9^ (P < 0.0001; Figure 3A).

We next asked whether these approaches could distinguish cancer-specific transcriptional programs, rather than differences in gene expression shared across tumors. APEX more accurately recovered genes preferentially expressed in each cancer type, whereas alternative approaches showed weaker separation of cancer-specific expression patterns (Figure 3B). Although deeper or targeted sequencing could improve the performance of individual approaches, these head-to-head comparisons show that integrating complementary chromatin and fragmentomic features with APEX provides substantially greater resolution of tumor gene expression than existing plasma-based methods.

### Noninvasive transcriptional subtyping with APEX

Accurate cancer subtyping is critical for diagnosis, prognosis, and treatment selection. We therefore asked whether APEX could recover subtype-specific transcriptional differences in a larger, independent plasma cohort and when applied to more widely available single-mark cfChIP-seq data. We analyzed 132 previously published H3K4me3 profiles spanning eight cancer subtypes (**Table S10**)^17^. Because H3K4me3 is the most widely profiled cfChIP-seq mark and H3K36me3 was not available in this cohort, we applied the H3K4me3 component of APEX to evaluate its utility in existing single-mark datasets. Subtype-specific expression was assessed using RNA-seq signatures derived from independent public datasets matched by tumor subtype (**Table S8**). Despite using a single histone mark, APEX–H3K4me3 significantly outperformed conventional promoter-aggregated H3K4me3 signal in distinguishing subtype-specific transcriptional programs (Figures 4A and 4B).

Because APEX outputs inferred gene expression, it enables the direct application of established RNA-seq-based analytical frameworks to plasma data. PurIST is a validated single-sample transcriptional classifier that assigns pancreatic tumors to prognostically distinct basal or classical subtypes based on the relative expression of a 16-gene signature, with important implications for patient outcomes and therapeutic sensitivity^29^. We applied PurIST to APEX-inferred gene expression, or to signal from individual histone marks, using plasma from 30 patients with pancreatic cancer whose subtypes had been independently established by tumor RNA-seq (**Table S11**). APEX correctly classified 18 of 22 classical tumors and 6 of 8 basal tumors, corresponding to an overall accuracy of 80% and a balanced accuracy of 78%. This performance exceeded classification using individual histone marks alone (balanced accuracy: H3K36me3 = 0.60; H3K4me3 = 0.63; Figures 4C and 4D). As expected, classification performance was higher among patients with detectable tumor fraction (>3% by ichorCNA; **Figure S17**). These results demonstrate that clinically relevant pancreatic cancer transcriptional subtypes can be measured noninvasively from plasma-inferred expression profiles.

### Noninvasive profiling of cell-surface therapeutic targets with APEX

Building on our previous finding that APEX-inferred DLL3 expression and SCLC subtype are associated with response to the DLL3-directed bispecific T-cell engager tarlatamab^30^, we asked whether APEX could more broadly resolve therapeutically relevant cell-surface expression in SCLC. Using baseline plasma from 34 patients prior to tarlatamab treatment, we identified the ten most subtype-enriched cell-surface genes for each SCLC transcriptional subtype. Twenty-seven of 30 (90%) genes showed concordant enrichment in independent tumor RNA-seq from 271 SCLC patients in the IMpower133 trial (empirical *P* = 0.002; Figure 5A)^31,32^. We next focused on CD276 (B7-H3), DLL3, SEZ6, and TACSTD2 (TROP2), which encode targets of therapies under clinical investigation in SCLC^33^. APEX similarly recapitulated subtype-associated expression patterns for these targets observed by tumor RNA-seq in IMpower133 (Figure 5B).

We next analyzed longitudinal plasma samples from patients treated with tarlatamab (**Table S12**). Serial APEX profiling revealed intra-patient shifts in dominant surface-target expression during therapy, with transitions among DLL3-, SEZ6-, and CD276-dominant states (Figure 5C). Among 20 patients with paired baseline and late on-treatment samples, CD276-dominant profiles increased from 20% (4/20) to 35% (7/20), consistent with the persistence of non-ASCL1 SCLC states during tarlatamab treatment^30^. Given the emerging clinical activity of B7-H3-directed antibody–drug conjugates in SCLC, we assessed B7-H3 protein expression in SCLC patient-derived xenografts and observed membranous staining across ASCL1-, NEUROD1-, and POU2F3-defined states (Figure 5D), consistent with previous findings^34^.

We next asked whether APEX could identify candidate therapeutic targets more broadly across cancers. We compiled APEX-inferred expression profiles from 451 patients across 24 cancer types analyzed in this study (**Table S15**). Using percentile-based expression thresholds analogous to those used to contextualize tissue RNA-seq measurements^35^, 204 of 451 patients (45%) had high inferred expression of at least one target of an FDA-approved expression-based therapy, including DLL3, ERBB2, F3, FOLR1, FOLH1, NECTIN4, MET, and TACSTD2 (Figure 5E). Target expression varied substantially within and across cancer types, including in cancers outside established therapeutic indications. In nine patients with matched tumor tissue, APEX-inferred DLL3 and TACSTD2 expression was associated with corresponding tumor protein expression by immunohistochemistry (Figure 5F). These findings demonstrate the potential of plasma gene-expression profiling to noninvasively resolve therapeutic target heterogeneity and track its evolution during treatment.

### Plasma-inferred NECTIN4 predicts response and survival to enfortumab vedotin

Emerging tissue-based studies have shown that tumor *NECTIN4* expression correlates with clinical response to enfortumab vedotin (EV), an antibody-drug conjugate targeting *NECTIN4* in metastatic urothelial carcinoma^36–39^. We therefore hypothesized that *NECTIN4* expression inferred from baseline plasma could noninvasively predict clinical outcomes in patients with metastatic bladder cancer treated with EV monotherapy. To test this, we performed cfChIP-seq on baseline plasma samples and inferred tumor gene expression using APEX (Figures 6A and 6B; **Table S13**).

APEX-inferred *NECTIN4* expression was significantly higher in responders (complete or partial response) compared to non-responders (stable or progressive disease) (*P* = 1.9 × 10^–3^; Figures 6C and **S18A–D**; **Table S14**). While 58% of patients with plasma-inferred *NECTIN4* expression above the cohort median objectively responded to EV, none with predicted expression below the median did, highlighting the negative predictive value of the assay (Figure 6D).

In contrast, *NECTIN4* estimates derived from individual histone marks showed weaker or nonsignificant associations with response. Plasma-inferred expression of other antibody-drug conjugate (ADC) targets not directly targeted by EV, including *ERBB2* and *TACSTD2*, tumor fraction, and *NECTIN4* amplification were not associated with treatment response, supporting the specificity of the *NECTIN4* signal (Figures 6C and **S18E–J**). Consistent with this finding, responders were enriched for urothelial luminal genes known to associate with *NECTIN4* expression and favorable outcomes^40^, while non-responders showed increased activation of epithelial-mesenchymal transition (EMT)-related genes that have been associated with worse outcomes^41^ (Figures 6C and **S18H**; **Table S8**).

Baseline APEX-inferred *NECTIN4* expression was also significantly associated with both progression-free survival and overall survival. This association was stronger than that observed using individual histone mark coverage, plasma tumor fraction, or *NECTIN4* amplification (Figures 6E, 6F, **S18J, S18K, and S19**). Multivariable Cox proportional hazards models confirmed that APEX-inferred *NECTIN4* remained independently associated with survival after accounting for tumor fraction, metastatic disease burden, line of therapy, and performance status, supporting its role as an expression-based biomarker for EV sensitivity (Figure 6G, **S18L, and S18M**).

## DISCUSSION

In this study, we show that complementary chromatin and fragmentomic features in plasma jointly encode tumor gene expression across regulatory regions. We develop APEX, a machine-learning framework for noninvasive inference of tumor gene expression from plasma that integrates chromatin and fragmentomic features from cfChIP-seq. By jointly modeling these features, APEX captures complementary aspects of transcriptional regulation that are inaccessible to either modality alone. Further, by leveraging more than 270,000 paired observations of gene expression and local chromatin features across the genome, APEX learns gene-agnostic relationships between epigenomic state and transcription, enabling robust inference in independent cohorts. Systematic screening of plasma-derived chromatin features revealed that proximal gene-body H3K4me3 signal is more informative of expression levels than aggregate promoter-proximal signal, consistent with prior work linking extended H3K4me3 occupancy to lineage-specific and oncogene-associated transcriptional programs^42^. In addition, H3K36me3 signal, particularly in the 3′ gene body and regions of maximal coverage, provided complementary predictive power, reflecting its association with transcriptional elongation^26^.

Beyond histone mark enrichment, APEX leverages fragmentomic features that reflect local chromatin structure. Notably, depletion of nucleosome-sized H3K36me3-immunoprecipitated fragments and relative enrichment of subnucleosomal and di-/trinucleosomal fragments were correlated with gene expression. These observations align with prior studies linking fragment length distributions to cancer-specific chromatin accessibility and mutation burden^9,43^, and suggest that fragment-length features can enhance inference of tumor-specific transcriptional activity beyond coverage-based measures alone. Importantly, library preparation approaches that disrupt native fragment length distributions, such as transposition-based assays^21^ or size selection, diminish fragmentomic signal and are suboptimal for gene expression prediction.

In contrast, H3K27ac and DNA methylation contributed little to gene-level expression prediction, even when enhancer–gene relationships and strict quality thresholds were applied^25^. This likely reflects a combination of redundancy with promoter- and gene-body-associated marks, greater signal degradation in plasma, and limitations of current enhancer-promoter annotations, which assume homogeneous cell populations and may not capture tissue- and gene-specific enhancer usage in heterogeneous cell-free chromatin mixtures. More precise context-specific enhancer-promoter modeling may improve gene expression prediction, although H3K27ac and DNA methylation remain well established for tumor subtyping and regulatory state classification^17^.

A key advantage of APEX is that it outputs inferred gene expression in a format directly compatible with standard RNA-seq analytical frameworks. This enables extension of established transcriptomic analyses, including differential expression, gene set enrichment, and expression-based classifiers, like PurIST^29^, to liquid biopsy data. Accordingly, we demonstrate improved cancer subtyping and more accurate recovery of lineage-specific transcriptional programs relative to individual histone marks. Notably, APEX was evaluated across a broad pan-cancer cohort encompassing 24 tumor types and 559 patient samples, representing, to our knowledge, the most diverse evaluation of plasma-based transcriptional inference across cancers to date and demonstrating its generalizability. APEX further showed robustness across input volumes, with successful application to cfChIP-seq data generation from 200 μL to 1 mL of plasma.

Our study has several limitations. Although APEX improves expression inference at lower tumor fractions relative to individual histone features, signal acquisition remains unstable at very low tumor fractions (<0.5%). Accordingly, our method is not intended for minimal residual disease detection or early cancer screening, but is most applicable in metastatic disease at diagnosis or progression, where median tumor fractions are closer to ~3%^22^. While expression predictions retain some dependence on tumor fraction, explicit linear adjustment risks removing biologically meaningful signal, and APEX predictions were empirically stable without tumor-fraction correction. As with other cfChIP-seq-based approaches, pre-analytical and technical variables, including sample handling, freeze–thaw cycles, and antibody performance, may influence data quality, although robust performance across multiple, diverse cohorts suggests resilience to such variation. In addition, APEX was trained using matched single-site RNA-seq as a reference, which may incompletely capture heterogeneity across metastatic lesions; nevertheless, robust performance under label perturbation suggests that APEX captures conserved transcriptional programs encoded in circulating tumor-derived chromatin.

Conceptually, APEX infers transcriptional activity from epigenomic features that reflect active gene regulation rather than direct protein measurements. For many therapeutic targets, transcriptional activity is a major determinant of protein expression, and concordance between plasma-inferred expression and tumor protein abundance was observed for selected targets by immunohistochemistry. However, this validation was limited in scope, and further evaluation on a target-by-target basis will be required to define the relationship between plasma-inferred transcription and tumor protein abundance. While applied to protein-coding genes, APEX can be extended to non-coding transcripts, although this will require further validation.

In metastatic bladder cancer, baseline APEX-inferred *NECTIN4* expression was associated with response and survival following EV, outperforming tumor fraction, *NECTIN4* amplification status, and individual histone mark coverage. Prior tissue-based studies have reported variable relationships between NECTIN4 expression and clinical outcomes^37–39,45^, potentially reflecting differences in treatment regimen^45^, spatial and temporal heterogeneity, protein localization^39^, and the lack of standardized antibodies or scoring methods^37^. Plasma-based inference may offer a complementary approach by providing a contemporaneous and potentially more systemic assessment of tumor expression without reliance on a single tissue biopsy. Consistent with the biological relevance of these measurements, APEX also recapitulated known biological programs associated with favorable and unfavorable outcomes^36,40,41^ and showed greater specificity for *NECTIN4* relative to other ADC targets. These findings were derived from a modest retrospective cohort and further validation in larger, prospective studies will be needed to determine the utility of plasma-inferred *NECTIN4* as a treatment-associated biomarker.

In our previous study, we applied APEX to longitudinal plasma samples from patients with SCLC treated with tarlatamab to study response and resistance^30^. APEX identified transcription-factor-defined states and transcriptional programs associated with treatment response, which were then validated in experimental models. Here, we extend this work to therapeutically relevant cell-surface targets. APEX identified subtype-associated differences in target expression and tracked changes in dominant target states during treatment. Consistent with prior findings, DLL3-dominant states decreased at later timepoints of tarlatamab treatment, likely reflecting their association with tarlatamab response^30,46^, whereas B7-H3 (CD276)-dominant profiles persisted. Notably, B7-H3 protein expression was observed across SCLC transcriptional subtypes as was previously observed using patient tissue^34^, suggesting a broader distribution than DLL3 and supporting its relevance as B7-H3-directed antibody–drug conjugates enter clinical development in SCLC. These results suggest that changes in SCLC transcriptional state are accompanied by changes in the surface-target landscape and may reveal alternative therapeutic vulnerabilities during treatment. Serial plasma profiling may therefore provide a way to study tumor evolution in patients and generate hypotheses for follow-up in experimental models.

This ability may become increasingly relevant as therapies directed against variably expressed cell-surface targets, including ADCs, multispecific antibodies, RLTs, and CAR-T cells, continue to expand^2^. Across cancer types, APEX identified high inferred expression of approved or investigational therapeutic targets in a substantial proportion of patients, including targets not routinely evaluated in the corresponding tumor types. These findings do not establish target protein abundance or therapeutic sensitivity, and the clinical value of expression-based treatment selection remains to be determined. However, plasma-inferred expression could help prioritize targets for tissue-based confirmation or provide an orthogonal measure when contemporary tissue is unavailable. This may be particularly useful in metastatic disease, where biomarker assessment often relies on tissue from a single site and time point despite spatial heterogeneity and continued evolution under therapy. With prospective and target-specific validation, longitudinal plasma expression profiling could complement tissue- and DNA-based biomarkers by providing a dynamic view of therapeutically relevant tumor expression.

## RESOURCE AVAILABILITY

### Lead contact

Requests for further information and resources should be directed to and will be fulfilled by the lead contact, Matthew L. Freedman (matthew_freedman@dfci.harvard.edu).

### Materials availability

This study did not generate new unique reagents.

### Data and code availability

#### Data

- De-identified human sequencing data and associated clinical metadata from the DFCI metastatic bladder cancer cohort (*n* = 24 patients) and DFCI metastatic pancreatic cancer cohort (*n* = 26 patients) are being deposited in dbGaP under controlled access (accession phs004572.v1.p1) and will be available upon publication. Due to privacy restrictions regarding genomic data, raw sequencing data from the MOSCATO–1, MOSCATO–2, SCLC tarlatamab cohorts, and the limit-of-detection dilution experiments, can be shared upon reasonable request under a data use agreement.
- Processed data derived from these cohorts (*n* = 1,274 epigenomic profiles; **Supplementary Table 15**), including bigWig coverage tracks, fragment-level BED files, and gene-level feature matrices, have been deposited at Gene Expression Omnibus (GEO). All datasets are available under accession GSE318228 using access token ansnycwantkdjaf, except for data from the SCLC tarlatamab cohort, which has been deposited in GSE324733 and can be accessed with the token odybukqifrgjlgd. These will be made publicly available as of the date of publication. APEX-inferred gene expression matrices for all cohorts are also provided as **Supplementary Data**. Raw and processed data from cfChIP-seq gene-body benchmarking analyses using mixed prostate cancer cell line and non-cancer donor samples (*n* = 84 epigenomic profiles), as well as LNCaP chromatin fragmentation comparisons between MNase- and Covarisbased protocols (*n* = 10 biological replicates per condition), have also been deposited in GEO (GSE318228).
- Raw bulk tumor RNA-seq data for patients enrolled in the MOSCATO–1 and MOSCATO–2 trials are available at the European Genome–Phenome Archive (EGA; EGAC00001002937). Raw and processed bulk tumor RNA-seq data for the metastatic pancreatic cancer cohort were obtained from a companion study^47^ and will be publicly available at the corresponding repository (accession phs004572.v1.p1). Processed tumor RNA-seq expression matrices used in this study are provided as **Supplementary Data**.
- The IMpower133 RNA-seq data^31,32^ (*n* = 271 patient profiles) were obtained through an MTA from Genentech by M.G.O.
- Publicly available datasets used for model training, benchmarking, or comparative analyses include bulk RNA-seq expression and matched clinical data from TCGA and GTEx accessed via the UCSC Xena Browser (https://xenabrowser.net/datapages/?cohort=TCGA%20TARGET%20GTEx; accessed June 7, 2025) and the TCGABiolinks R package (v2.36.0)^48^; epigenomic and transcriptomic reference datasets from the Roadmap Epigenomics Project (https://egg2.wustl.edu/roadmap/web_portal/processed_data.html); small-cell lung cancer RNA-seq data from George et al. (Nature, 2015; PMID: 26168399; Supplementary Table 10)^49^; metastatic prostate cancer RNA-seq datasets from the Prostate Cancer Atlas, including the “WCDT-MCRPC” and “T.C.B. 2015” studies (https://www.prostatecanceratlas.org/api/files/public/gc_txi.abundance.fst; dbGaP accession phs000909.v1.p1); and control LNCaP RNA-seq data from GEO (GSE128749). A curated list of candidate cancer cell-surface targets was obtained from Hu et al. (Nature Cancer, 2021; PMID: 35121907; Supplementary Table 9)^50^.
- Public genomic and epigenomic annotations were obtained from ENCODE genomic exclusion regions (ENCFF001TDO; https://www.encodeproject.org/files/ENCFF001TDO/@@download/ENCFF001TDO.bed.gz), the Boyle Lab hg19 blacklist (https://github.com/Boyle-Lab/Blacklist/blob/master/lists/hg19-blacklist.v2.bed.gz), reference genome coordinates from the BSgenome.Hsapiens.UCSC.hg19 R package (v1.4.3), protein-coding gene annotations from GENCODE (GRCh37, release 27), enhancer annotations from GeneHancer (v5.22, GRCh37)^25^, DNase I hypersensitive site annotations (https://zenodo.org/record/3838751/files/DHS_Index_and_Vocabulary_hg19_WM20190703.txt.gz), genome-wide ChromHMM 18-state annotations across 833 biosamples from the Epimap project (hg19; https://personal.broadinstitute.org/cboix/epimap/ChromHMM/observed_aux_18_hg19/CALLS/), and public ChIP-seq and paired RNA-seq datasets from the Roadmap Epigenomics Project (https://egg2.wustl.edu/roadmap/web_portal/processed_data.html).
- Any additional processed datasets generated in this study will be shared by the lead contact upon request.

#### Code

- Preprocessing of cfChIP-seq data was performed using the Nextflow-based SNAPIE pipeline (v1.5.1), available at https://github.com/prc992/SNAPIE^51^.
- EPIC-seq data were processed using the EPIC-seq framework (https://epic.stanford.edu)^9^.
- APEX (v4.3.0) is available as an R package at github.com/Baca-Lab/APEX and is freely available for non-profit academic research under a non-commercial license. The package provides functions for cfChIP-seq quality assessment, extraction of epigenomic and fragmentomic features, genome-wide gene-expression inference using pretrained APEX models, differential expression and gene-set analyses, and data visualization, including interactive plots. A reproducible Snakemake^52^ workflow is also provided for scalable execution on high-performance computing systems. Documentation, vignettes, and example input files are included to facilitate implementation and reproducibility.

## ONLINE METHODS

### Human Participants

All processing of plasma samples for cfDNA and cfChIP-seq assays was performed under Dana-Farber/Harvard Cancer Center IRB protocol 21–764, in accordance with institutional guidelines, HIPAA regulations, and the Declaration of Helsinki. For all clinical studies, patients provided written informed consent for genomic profiling and data sharing for research purposes. Plasma and tissue samples were collected under IRB-approved protocols for metastatic pancreatic cancer (DFCI 03–189) and metastatic urothelial cancer (DFCI 02–021) patients. IRB approvals for the previously published study from Baca et al.^17^ and the MOSCATO trials^23^ are described in the original publications. Additional cohort-specific details are provided below and in **Tables S3, S7, S10, S11, S12, and S13**. Laboratory personnel processing plasma samples were blinded to clinical information and study outcomes. Clinical data curation and computational analyses, including APEX model generation and subtype classification, were performed independently of plasma processing.

### MOSCATO Pan-Cancer Clinical Cohort

Patients aged 18 years or older with unresectable or metastatic cancer who had progressed after at least one prior line of therapy or had limited treatment options and at least one accessible biopsy site were enrolled in the Molecular Screening for Cancer Treatment Optimization (MOSCATO) clinical trial (NCT01566019)^23^, a monocentric, prospective study conducted at Gustave Roussy. The study consisted of two sequential phases: MOSCATO–1 (2011–2016) and MOSCATO–2 (2017–2020).

Patients from MOSCATO–1 with matched plasma and tumor RNA-seq data were assigned to the training cohort, whereas patients from MOSCATO–2 were assigned to the test cohort. All participants provided written informed consent for trial participation, genomic and transcriptomic profiling, and data sharing for research purposes. The protocol was approved by the Institutional Review Board of Gustave Roussy and conducted in accordance with the Declaration of Helsinki and Good Clinical Practice guidelines.

### Metastatic Urothelial Cancer Cohort

Patients with metastatic bladder cancer were identified from the Dana-Farber Cancer Institute (DFCI) Arthur and Linda Gelb Center for Translational Research biobank and associated clinical database under IRB-approved DFCI protocol 02–021. All samples and clinical data were de-identified prior to analysis.

Eligible patients had histologically confirmed urothelial carcinoma of the bladder with metastatic or unresectable disease and were treated with enfortumab vedotin monotherapy. Only patients aged 18 years or older were included. Exclusion criteria included prior exposure to NECTIN4-targeting therapies and receipt of concurrent systemic anticancer therapies in combination with enfortumab vedotin.

Plasma samples were required to be collected either within 90 days prior to initiation of enfortumab vedotin without interim cancer-directed therapy or within 8 days following treatment initiation (median [IQR], 0 [0–2] days). Five of 24 plasma samples were collected after treatment initiation (day 7 or 8). For each patient, a single aliquot of 810 μL biobanked plasma was obtained. Plasma samples had been processed and stored at −80 °C and had not undergone any prior freeze–thaw cycles before analysis.

Radiographic response assessments were performed by a trained clinician blinded to plasma-based analyses in accordance with RECIST version 1.1 criteria. Best overall response was determined based on changes in the sum of target lesion measurements. Clinical variables, including histologic diagnosis, treatment response, metastatic distribution, Karnofsky performance status, number of prior lines of therapy, and dose reductions prior to best response, were extracted from the electronic medical record by a trained clinician blinded to plasma-based analyses. Technical batch information for plasma processing was recorded.

Plasma tumor fraction was inferred from cfDNA sequencing data using ichorCNA (v0.2.0)^24^. Default parameters were used except for 500 kb bins to enable resolution of focal copy-number alterations encompassing the *NECTIN4* locus.

### Metastatic Pancreatic Cancer Cohort

Plasma cfChIP-seq profiles from patients with metastatic pancreatic ductal adenocarcinoma were obtained from two sources. The PancSeq study included samples collected at Dana-Farber Cancer Institute (DFCI) under IRB protocol 03–189 (*n* = 26)^47^. Additional plasma samples were collected at Gustave Roussy (*n* = 4) under institutional review board-approved protocols described in the respective study documentation.

Only samples with matched H3K4me3 and H3K36me3 cfChIP-seq profiles and paired bulk tumor RNA-seq were included in subtype analyses (*n* = 8 basal; *n* = 22 classical). One sample (M2271) was also included in the MOSCATO–2 validation cohort and was analyzed consistently across datasets.

### Small-cell lung cancer cohort

Plasma samples were prospectively collected from patients with de novo or transformed small-cell lung cancer (SCLC) treated with tarlatamab at Dana-Farber Cancer Institute between September 2024 and December 2025. The full clinical cohort is described in the manuscript by Vasseur et al. (2026)^30^. Analyses of treatment response, longitudinal dynamics, and SCLC subtype were restricted to patients with SCLC, excluding those with large-cell neuroendocrine carcinoma or carcinoid tumors. The final analytic cohort comprised 34 patients, of whom 18 derived clinical benefit, defined as stable disease or partial response, and 16 had progressive disease and were classified as having no clinical benefit. In total, 109 longitudinal plasma samples were collected across baseline and on-treatment time points, including cycle 1 day 8, cycle 1 day 15, cycle 2 day 1, cycle 2 day 15, and cycle 3 day 1.

### Healthy volunteer cohort

Plasma samples were collected from 15 individuals without a known cancer diagnosis between September 2025 and June 2026. Participants were recruited through Dana-Farber Cancer Institute and the Mass General Brigham Biobank and provided informed consent under Dana-Farber Cancer Institute protocol 01–130 or Mass General Brigham Biobank protocol 2009P002312.

### Cell Lines

LNCaP cells (ATCC CRL-1740) were purchased from ATCC, authenticated by short tandem repeat (STR) profiling, and confirmed to match the parental LNCaP line in the ATCC database. Cells were tested for mycoplasma contamination using the LookOut Mycoplasma PCR Detection Kit (Sigma-Aldrich, #D9307) prior to experimentation. LNCaP cells were maintained in RPMI medium supplemented with 10% fetal bovine serum and antibiotics at 37 °C in 5% CO_2_. No genetic modifications were introduced in this study.

### Cell-free chromatin immunoprecipitation sequencing (cfChIP-seq)

Antibodies were covalently conjugated to Dynabeads M-270 Epoxy (Invitrogen, 14302D) using the Dynabeads Antibody Coupling Kit (Invitrogen, 14311D) according to the manufacturer’s instructions, with 5 μg of antibody coupled to 1 mg of beads for at least 16 h at 37 °C with rotation. After coupling, beads were stored at 50 μg ml^−1^ at 4 °C and pre-cleared in 0.1% BSA in PBS for 5 min at 4 °C prior to use. For chromatin immunoprecipitation, up to 810 μl of thawed plasma was used; samples with lower input volumes were brought to 810 μl with chromatin-preserving buffer (50 mM Tris-HCl pH 7.6, 1 mM CaCl_2_, 0.2% NP-40, 5 mM sodium butyrate, complete protease inhibitor cocktail, and 0.5 mM phenylmethylsulfonyl fluoride). Samples were supplemented with protease inhibitor cocktail (Roche, 11873580001), centrifuged at 3,000g for 15 min at 4 °C to remove residual debris, and combined with 90 μl of salt–detergent buffer to solubilize cell-free chromatin. The resulting 900 μl of plasma was incubated with 1 μg of antibody-coupled magnetic beads, with sequential immunoprecipitation against H3K27ac (6 h), H3K36me3 (overnight), and H3K4me3 (3 h) performed on the same plasma sample at 4 °C with rotation; for gene-body benchmarking analyses, all gene body–directed antibodies were incubated overnight. Antibody details are provided in **Table S3**.

Immunoprecipitated chromatin was preserved in chromatin-preserving buffer prior to washing. Beads were washed sequentially three times each with 150 μl of low-salt wash buffer (0.1% SDS, 1% Triton X-100, 2 mM EDTA, 150 mM NaCl, 20 mM Tris-HCl pH 7.5), 150 μl of high-salt wash buffer (500 mM NaCl), and 150 μl of LiCl wash buffer (250 mM LiCl, 1% NP-40, 1% sodium deoxycholate, 1 mM EDTA, 10 mM Tris-HCl pH 7.5), followed by a final rinse in TE buffer. Chromatin was eluted and reverse-crosslinked in DNA extraction buffer (0.1 M NaHCO_3_, sodium deoxycholate, 1 mM EDTA, 10 mM Tris-HCl pH 7.5), followed by a final rinse in TE 1% SDS) supplemented with Proteinase K (0.6 mg ml^−1^; QIAGEN, 19131) and RNase A (0.4 mg ml^−1^; Thermo Fisher Scientific, 12091021) by sequential incubation at 37 °C for 10 min, 50 °C for 1 h, and 65 °C for 90 min. DNA was purified using the ChIP DNA Clean & Concentrator kit (Zymo Research, #D5205). Sequencing libraries were prepared using the ThruPLEX DNA-Seq kit (Takara Bio, R400675), purified with AMPure XP beads (Beckman Coulter, A63880), assessed for size distribution using an Agilent 2100 Bioanalyzer with a High Sensitivity DNA chip (Agilent, 5067–4626), and sequenced as 150-bp paired-end reads on the Illumina NovaSeq X Plus platform (Novogene).

### Assessment of DNA methylation features for plasma-based gene expression

Methylated DNA immunoprecipitation and sequencing (cfMeDIP-seq) was performed as previously described to assess whether genome-wide 5-methylcytosine profiles could further improve APEX performance^17,53^. We generated cfMeDIP-seq profiles for 18 samples from the prospective validation cohort, each of which already had matched cfChIP-seq data for H3K4me3, H3K27ac and H3K36me3. To quantify the incremental predictive value of methylation, we trained APEX models with and without cfMeDIP-derived features and evaluated performance using leave-one-out cross-validation across these 18 samples. Inclusion of cfMeDIP-seq features yielded a statistically significant but modest improvement in RMSE relative to the model without cfMeDIP-seq (unpaired Wilcoxon test, *P* = 0.024; median RMSE: 1.49 vs. 1.51). Given the small effect size, additional cost, and increased experimental complexity associated with cfMeDIP-seq, methylation features were not incorporated into the final APEX model (Methods, “Development of APEX”).

### Cell-free DNA isolation and low-pass whole genome sequencing (LP-WGS)

Cell-free DNA was isolated from 1 mL of plasma using the Circulating Nucleic Acids Kit (QIAGEN, 55114), eluted in AE buffer, and stored at −80 °C. Sequencing libraries were prepared using the ThruPLEX DNA-Seq kit (Takara Bio, R400675) according to the manufacturer’s instructions and purified with AMPure XP beads (Beckman Coulter, A63880). Libraries were sequenced on an Illumina NovaSeq X Plus system (Novogene) with 150-bp paired-end reads at approximately 1–2× genome-wide coverage. Copy-number profiles and plasma tumor fraction were inferred using the ichorCNA R package (v0.2.0)^24^, which estimates tumor-derived DNA content based on read depth across genomic bins using default parameters.

### Processing of cell-free ChIP-seq and DNA sequencing data

FASTQ files were demultiplexed with bcl2fastq2 (RRID:SCR_015058; v2.19) to generate sample-specific files based on unique barcode sequences. All subsequent preprocessing steps were performed using a reproducible Nextflow-based pipeline (SNAPIE v1.5.1) optimized for cfChIP-seq analysis (https://github.com/prc992/SNAPIE)^51^. Briefly, paired-end sequencing reads were adapter-trimmed using Trim Galore (v0.6.10)^54^ (trim_method set to TRIM_GALORE), but quality trimming was disabled (--quality 0) to preserve fragment length distributions and end-motif sequences. Trimmed reads were aligned to the hg19 reference genome (GCA_000001405.1) using Burrows-Wheeler Aligner (v0.7.18)^55^ with extended insert size modeling (–I 250,200,2000,10) to prevent misclassification of short and long cfDNA fragments as improperly paired. Mapped reads were filtered to retain uniquely aligned reads and sorted by coordinates using samtools (v1.15.1). PCR and optical duplicates were removed using Picard MarkDuplicates (v2.27.4; RRID:SCR_006525). Low-quality reads were removed using samtools (v1.15.1) with flags –f 3 –F 3844 –q 30 to retain only properly paired, high-confidence (mapping quality ≥ 30) reads, while excluding unmapped, secondary, QC-failed, duplicated, and supplementary reads.

The resulting BAM files were converted into BEDPE format using BEDtools (v2.30.0)^56^ bamtobed with the –bedpe flag and fragments with non-negative lengths were retained. Fragments overlapping with regions in the ENCODE hg19 exclusion list (ENCFF001TDO) were removed. The resulting fragment files from SNAPIE were imported in R as GRanges objects (GenomicRanges v1.56.2)^57^ and extremely long (>500 bp) and short (<20 bp) fragments were removed for downstream analysis. Coverage tracks were generated from aligned BAM files using bamCoverage (deepTools v3.5.6^58^) with a 10-bp bin size and RPKM normalization, and exported in bigWig format.

### Enrichment score calculation and quality control

To assess signal-to-noise quality of cfChIP-seq libraries, we computed enrichment scores by comparing chromatin signal over genomic regions where each histone modification is expected to occur (“on-target”) versus regions where it should be depleted (“off-target”). Genome-wide 18-state ChromHMM annotations across 833 biosamples were obtained from the Epimap project (hg19; (https://personal.broadinstitute.org/cboix/epimap/ChromHMM/observed_aux_18_hg19/CALLS/)^59^) and partitioned into 200-bp bins. For each histone mark, on-target regions were defined as bins annotated with mark-associated ChromHMM states in >50% of biosamples (H3K27ac: 1_TssA, 3_TssFlnkU, 8_EnhG2, 9_EnhA1; H3K4me3: 1_TssA, 2_TssFlnk, 3_TssFlnkU, 4_TssFlnkD, 8_EnhG2, 14_TssBiv; H3K36me3: 5_Tx, 7_EnhG1, 8_EnhG2). Off-target regions were defined as bins lacking any corresponding on-target ChromHMM states. ENCODE blacklist regions (https://github.com/Boyle-Lab/Blacklist/blob/master/lists/hg19-blacklist.v2.bed.gz)^60^, chrX, and chrY were excluded, and off-target regions within 10 kb of on-target regions were removed. Remaining regions were merged and filtered to retain intervals ≥1 kb, yielding 11,530/219,810 (H3K27ac), 13,795/281,427 (H3K4me3), and 12,721/90,269 (H3K36me3) on-/off-target regions, respectively. To quantify immunoprecipitation (IP) efficiency and background noise, we calculated enrichment scores as the ratio of normalized fragment coverage in on-target versus off-target regions. For each sample and histone mark, the enrichment score was defined as:

ES=RonBonRoffBoff,

where $R_{\text{on}}$ and $R_{\text{off}}$ represent fragment counts in on-target and off-target regions, respectively, while $B_{\text{on}}$ and $B_{\text{off}}$ denote the corresponding genomic lengths.

For the training and test cohort (MOSCATO–1 and MOSCATO–2 cohorts), samples were required to meet quality thresholds of enrichment score >7 H3K4me3 and H3K27ac, and >2 for H3K36me3, with a minimum of 1 million uniquely mapped fragments per sample. For all other cohorts, no strict quality threshold was applied unless otherwise stated.

### Plasma sample collection and processing in the MOSCATO cohorts

Plasma samples from the MOSCATO–1 and MOSCATO–2 clinical trials were collected on the same day as the tissue biopsy, corresponding to the participant’s inclusion date in the clinical trial^23^. Blood samples (3–10 mL) were drawn into EDTA tubes (BD Vacutainer, Beckton Dickinson and Company), then centrifuged at 1,000 × *g* for 10 minutes within 4 hours of collection. The resulting supernatant, containing the plasma, was subsequently centrifuged at 14,000 × *g* for 10 minutes at room temperature. The plasma was then stored at −80°C until further analysis.

### Tumor biopsy collection and pathological assessment in the MOSCATO cohorts

Needle biopsies were obtained as described in Pradat et al., *Cancer Discovery*, 2023^23^ from a metastatic lesion and immediately frozen in liquid nitrogen. A senior pathologist evaluated tumor cellularity on the hematoxylin and eosin-stained slide from the biopsy used for DNA and RNA extraction, which are reported in **Tables S1 and S7**.

### RNA library preparation and sequencing in the MOSCATO cohorts

Tumor RNA was extracted as described in Pradat et al., *Cancer Discovery*, 2023^23^, using the AllPrep DNA/RNA Mini Kit (QIAGEN, 80204) following the manufacturer’s instructions. RNA libraries were then prepared using the TruSeq Stranded mRNA kit (Illumina, #20020594), adhering to the supplier’s guidelines. PolyA-containing mRNA molecules were purified from 1 μg of total RNA using oligo(dT)-coupled magnetic beads with the Magnetic mRNA Isolation Kit (NEB, S1550S). RNA fragmentation was performed using divalent cations at elevated temperatures to generate 300 to 400 bp fragments. This was followed by double-strand cDNA synthesis, Illumina adapter ligation, and PCR amplification of the cDNA library for sequencing. Paired-end sequencing was conducted on the Illumina NextSeq 500 (75 bp).

### RNA-sequencing processing and gene expression quantification in the MOSCATO cohorts

Tumor RNA-sequencing data were processed as described in Pradat et al., *Cancer Discovery*, 2023^23^, using the reproducible Snakemake workflow available at https://github.com/gustaveroussy/MetaPRISM_Public, which implements the pipeline described in https://github.com/gevaertlab/RNASeq_pipeline. Raw FASTQ files were preprocessed with Trim Galore (v0.4.4)^54^ to remove adapter sequences and low-quality bases. Transcript-level quantification was performed with Kallisto (v0.44.0)^61^ using the GENCODE v27 reference transcriptome (FASTA and GTF files; ftp.ebi.ac.uk/pub/databases/gencode/Gencode_human/release_27/).

Transcript-level estimates were summarized to gene-level counts using TxImport (v1.16.0)^62^, yielding expression estimates for 58,288 Ensembl genes. Gene identifiers were converted to gene symbols using the GENCODE v27 GTF annotation (GRCh37 lift-over), and multiple transcript entries mapping to the same gene were aggregated by summation. After filtering to genes with non-zero expression across all samples, 18,351 genes remained for analysis.

To account for sequencing depth and gene length, raw read counts were normalized to transcripts per kilobase million (TPM).

The TPM for gene j in sample k was calculated as:

$${\text{TPM}}_{j,k}=\frac{C_{j,k}/L_{j}}{\sum_{n=1}^{N}\left(C_{n,k}/L_{n}\right)}\times 10^{6},$$

where $C_{j,k}$ is the raw read count for gene j in sample $k,L_{j}$ is the gene length in kilobases (kb), and N is the total number of genes quantified in that sample. To stabilize variance for downstream comparative analyses, values were log_2_-transformed with a pseudocount of 1:

$${\text{Expression}}_{j,k}={\text{log}}_{2}\left({\text{TPM}}_{j,k}+1\right).$$

### Reference epigenomic analyses and benchmarking of gene-body histone mark antibodies

#### Calculating percent of human genome marked by histone modifications

To assess genome-wide coverage of gene body histone marks, we analyzed publicly available ChIP-seq datasets (*n* = 56) from the Roadmap Epigenomics Project^63^. These included H3K36me3 (*n* = 56), H3K79me1 (*n* = 5), H3K79me2 (*n* = 10), H3K9me1 (*n* = 3), and H4K20me1 (*n* = 11), with paired H3K4me3 (*n* = 56) and H3K27ac (*n* = 48) profiles for comparison (**Figure S1A**). Narrow peak calls for H3K4me3 and H3K27ac and broad peak calls for the gene body marks were downloaded from the Roadmap data portal (https://egg2.wustl.edu/roadmap/web_portal/processed_data.html). The hg19 reference genome coordinates were obtained using the BSgenome.Hsapiens.UCSC.hg19 R package (v1.4.3). To ensure mappability, regions prone to artifacts, including the ENCODE hg19 blacklist (ENCFF001TDO), genomic gaps (e.g., centromeres, telomeres), fix patches, and alternative haplotypes from the ‘Feb. 2009 GRCh37/hg19’ assembly, were excluded. Genome-wide coverage for each histone mark was calculated as the total width of non-overlapping peaks divided by the mappable genome size (2.92 × 10^9^ bp).

#### Association between histone ChIP-seq and RNA-seq

Publicly available ChIP-seq and paired RNA-seq datasets (*n* = 56) from the Roadmap Epigenomics Project^63^ were downloaded from https://egg2.wustl.edu/roadmap/web_portal/processed_data.html and analyzed to assess the relationship between histone marks at promoters, intergenic enhancers, intragenic enhancers, and gene bodies and RNA transcription (**Figure S1B**). Promoter regions were defined as ±3 kb from the transcription start site (TSS) using GENCODE v27 annotation lifted to the hg19. Gene body regions were defined as the region from the TSS to the transcription termination site (TTS). Enhancer regions were identified using the GeneHancer database (v5.22)^25^, selecting only high-confidence enhancers (regulatory_element_type = “Enhancer” and is_elite = 1). Coverage was computed by counting the number of aligned sequencing reads from the ChIP-seq tagAlign files that overlapped each defined region (promoter, gene body, or enhancers) using the countOverlaps function in GenomicRanges (R v1.56.2)^57^. For enhancers (intergenic and intragenic), coverage was averaged across all associated enhancers, weighted by the element–gene association score (combined_score).

ChIP-seq coverage was normalized to account for region length, e.g. gene-body signal by gene length, enhancer signal by the mean length of associated enhancers weighted by their GeneHancer combined_score, and promoter signal by a fixed width of 6 kb centered on the TSS. RNA-seq read counts (“57epigenomes.N.pc.GENESYMBOL.txt”; sample “E000”, representing Universal Human Reference RNA, was excluded) were normalized to transcripts per million (TPM) by dividing raw counts by gene length, scaling by the total read count per sample, and multiplying by one million. Only genes annotated in all relevant datasets (e.g. RNA-seq and GeneHancer v5.22 for enhancers; RNA-seq and GENCODE v27 annotations for promoters and gene bodies) were included in the analysis. Spearman correlations were computed between histone mark signals at promoters, gene bodies, and enhancers and TPM-normalized gene expression.

#### Benchmarking gene body histone mark antibodies for cfChIP-seq

We benchmarked antibodies targeting gene body-associated histone modifications to assess their utility for cell-free chromatin immunoprecipitation sequencing (cfChIP-seq) in plasma. Antibodies against H3K36me3, H4K20me1, H3K9me1, H3K79me1, H3K79me2, and H3K27me1 were selected based on prior usage in the literature, commercial availability, and reported validation (**Table S3**). Antibodies were conjugated to epoxy magnetic beads and cfChIP-seq was performed as described above in “Cell-free chromatin immunoprecipitation (cfChIP-seq).” Sequenced libraries were processed as described in “Processing of cell-free ChIP-seq and DNA sequencing data”. Read-level coverage over regions of interest was calculated by counting aligned fragments overlapping gene body regions using the countOverlaps function in GenomicRanges (R v1.56.2)^57^, normalized for gene length. As a positive control, each antibody was also tested in 1 μg fixed LNCaP chromatin, which was prepared as previously described.

We benchmarked antibody performance according to three criteria:

1. Number of unique fragments: The number of deduplicated, uniquely aligned fragments recovered from cfChIP-seq libraries after sequencing.
2. Gene body enrichment score: To assess immunoprecipitation specificity, we calculated the enrichment of recovered fragments in gene-body associated chromatin states (“Tx”, “EnhG1”, “EnhG2”) as described in “Enrichment score calculation and quality control”. As a negative control, we used enrichment estimates from low-pass whole-genome sequencing (WGS) data from the 15 samples in the training cohort, which are expected to show minimal gene body coverage bias (expected enrichment score ≈ 1).
3. Prostate-cancer-specific signal recovery: To evaluate signal specificity for disease-relevant transcription, we compared cfChIP-seq gene-body signal in plasma from patients with prostate cancer to that from non-cancer donors. For each sample, we computed the mean log_2_-normalized gene-body signal (normalized by gene length) across the top 500 prostate-specific genes and subtracted the mean log_2_-normalized signal across the top 500 white blood cell (WBC)-specific genes (see “Defining cancer- and normal-subtype-specific gene signatures” and **Table S8**). This difference corresponds to the log_2_ ratio of prostate-selective cfChIP-seq signal. We compared these prostate-selective values between antibody- and operator-matched prostate cancer and non-cancer plasma samples to identify antibodies that maximized recovery of tumor-specific signal.

#### Experimental comparison of H3K36me3 and H3K79me2 cfChIP-seq

Because H3K79me2 showed the strongest and most consistent association with transcription across cell-line and tissue ChIP-seq datasets (**Figs. S1A and S1B**), we sought to determine whether its limited recovery in plasma was specific to the initial prostate cancer samples. H3K36me3 and H3K79me2 cfChIP-seq were therefore performed in parallel using matched 1-mL plasma aliquots from five additional patients with bladder cancer, breast cancer, neuroendocrine prostate cancer, small cell lung cancer, or non-small cell lung cancer. Immunoprecipitations used 1 μg of either a polyclonal H3K36me3 antibody (Diagenode, C15410192) or a rabbit polyclonal H3K79me2 antibody (Abcam, ab3594) (**Table S3**). Each antibody was also evaluated using 1 μg of fixed LNCaP chromatin as a positive control. Libraries were generated, sequenced, and processed as described in “Cell-free chromatin immunoprecipitation (cfChIP-seq)” and “Processing of cell-free ChIP-seq and DNA sequencing data.”

Gene-body enrichment was visualized at the ACTB locus using genome-browser tracks and quantified using the enrichment-score framework described in “Enrichment score calculation and quality control,” comparing coverage in gene body-associated chromatin states with coverage in background regions located more than 10 kb from annotated gene bodies. Unique fragment counts were calculated after alignment, filtering, and deduplication. Enrichment scores and unique fragment recovery were compared between matched H3K36me3 and H3K79me2 plasma libraries using paired two-sided t-tests.

### Defining cancer- and normal-subtype-specific gene signatures

#### Data acquisition and preprocessing

RNA-seq expression data for The Cancer Genome Atlas (TCGA) and Genotype-Tissue Expression (GTEx) projects were obtained from the UCSC Xena Browser (https://xenabrowser.net/datapages/?cohort=TCGA%20TARGET%20GTEx; accessed June 7, 2025). The datasets included 10,535 TCGA tumor samples and 7,792 GTEx normal samples, provided in transcripts per million (TPM) units with matched phenotype annotations. Additional clinical and histopathologic annotations were retrieved using the R package TCGABiolinks (v2.36.0)^48^. These metadata were used to further subclassify breast cancers into ER-positive or ER-negative groups based on the er_status_by_ihc field, and esophageal cancers into adenocarcinoma or squamous cell carcinoma subtypes based on the primary_diagnosis field.

RNA-seq data for small-cell lung cancer (SCLC; *n* = 81) were obtained in log(FPKM + 1) format from Supplementary Table 10 of George *et al*., *Nature*, 2015^49^. Values were unlogged, the pseudocount of 1 was subtracted, and the remaining FPKM values were converted to TPM by dividing each gene’s FPKM by the total FPKM per sample and multiplying by one million. To supplement the largely localized prostate cancers represented in TCGA, RNA-seq data (in TPM units) for metastatic prostate cancer subtypes, neuroendocrine prostate cancer (NEPC; *n* = 15) and androgen receptor–pathway active prostate cancer (ARPC; *n* = 129), were obtained from the “Prostate Cancer Atlas” (https://www.prostatecanceratlas.org/api/files/public/gc_txi.abundance.fst), specifically from the “WCDT-MCRPC”^64^ and “T.C.B. 2015” (dbGaP study accession: phs000909.v1.p1) datasets.

Gene symbols were harmonized across all datasets using the HGNChelper R package (v0.8.15)^65^. All expression matrices in TPM units were merged on intersecting gene symbols, rescaled to a total of one million transcripts per sample, and subsequently log_2_ (TPM + 1) transformed. The resulting unified matrix comprised 18,551 samples spanning 91 phenotypes, including 38 cancer types and 53 normal tissues.

#### Derivation of cancer- and normal-specific gene signatures

Cancer- and normal-specific gene signatures were derived from the unified TCGA–GTEx–Prostate Cancer Atlas–SCLC dataset described above. For each cancer type, cancer-specific genes were defined as those significantly enriched (log_2_ fold change > 1; *P*-value adjusted for multiple hypothesis testing by the Benjamini-Hochberg method < 0.01) relative to (i) all other TCGA cancer types and (ii) all normal phenotypes in GTEx. Likewise, normal-specific genes were defined by comparing each normal tissue type to (i) all other GTEx normal tissues and (ii) all TCGA cancers using the same thresholds. Up to 500 overlapping differentially expressed genes (DEGs) shared between the two respective comparisons were retained as the final signature for each tissue or cancer type.

For analyses presented in Figure 3K, 4A, and 4B, the most specific genes among the cancers examined were determined using a specificity scoring framework. For each gene, the mean expression within each sample group was first computed. The specificity score for a given gene j in group g was defined as the difference between its mean expression in that group $\left({\overset{‾}{E}}_{j,g}\right)$ and the maximum mean expression in all other groups:

$${\text{Specificity}}_{j,g}={\overset{‾}{E}}_{j,g}-\underset{h\neq g}{\text{max}}\left({\overset{‾}{E}}_{j,h}\right).$$

Genes were ranked within each group by their specificity scores, and the top 10 most specific genes per group were selected. The final gene list comprised the union of all group-specific top 10 genes.

All gene signatures used in this study are enumerated in **Table S8**.

### In silico screen for cell-free chromatin features associated with gene expression

To systematically identify cfChIP-seq–derived chromatin features predictive of transcriptional activity, we performed a genome-wide *in silico* correlation screen between cell-free chromatin features and matched tumor RNA-seq data (**Table S5**).

Features were extracted as described under “Developmental of APEX: Feature extraction” (Methods) using default parameters. Promoter, gene body, intragenic enhancer, and intergenic enhancer regions were defined as described under “Development of APEX: Promoter, gene body, and enhancer reference” and each were divided into five equally sized bins, yielding 3,516 total features per gene. In specific instances requiring higher resolution for visualization (Figures 1E and **S3A–S3E)**, each genomic region was subdivided into 40 equally sized bins. Alternate promoter positions were adjusted as described under “Development of APEX: Selection of alternate promoters”.

Gene expression values were derived from log_2_(TPM + 1)-transformed RNA-seq data in the training cohort (*n* = 15). Genes with zero expression across all samples or zero evaluable fragments for any histone mark in any sample were excluded, resulting in 17,950 genes. Pairwise single-gene level Spearman correlations between each chromatin feature and gene expression value were computed using the cor() function in base R (v4.4.2) with parameters method = “spearman” and use = “pairwise.complete.obs”, such that correlations were calculated using all complete observation pairs.

### Associations between chromatin fragment length and gene expression

#### Experimental context and data sources

Associations between chromatin fragment length and gene expression were evaluated across three contexts: (1) cell-free chromatin immunoprecipitated fragments, (2) non-immunoprecipitated plasma cfDNA, and (3) MNase- or Covaris-digested LNCaP chromatin. For (1), cfChIP-seq was performed using antibodies to H3K4me3, H3K27ac, and H3K36me3, with low-pass whole-genome sequencing (LPWGS) as a negative control. For (2), cfDNA from prostate (M2594; 17.5 ng) and colorectal (M2673; 20 ng) cancer patients from the MOSCATO–2 clinical trial were sequenced on the Ultima Genomics UG100 platform using XGen adapters. The mean genome coverage was 120× for M2594 and 138× for M2673 and the mean read length was 171 bp and 178 bp, respectively. For (3), ChIP-seq libraries were prepared as described under “Cell-free chromatin immunoprecipitation sequencing (cfChIP-seq)” using 0.8–1 μg of MNase-digested (“MNase-digested chromatin preparation”) or Covaris-sheared (“Covaris-sheared chromatin preparation”) LNCaP chromatin.

#### Fragment length binning and correlation with gene expression

For each gene, fragment counts were computed across genomic regions (for example, gene bodies; Figure 2E) in 10-bp bins spanning fragment lengths of 20–500 bp. Bin frequencies were normalized by the total number of fragments per region. For each fragment-length bin, Spearman correlations were computed between fragment-length frequency and matched tumor RNA-seq expression across all genes using the cor() function in base R (v4.4.2) with parameters method = “spearman” and use = “pairwise.complete.obs”, such that correlations were calculated using all complete observation pairs.

Matched bulk RNA-seq data for the cfChIP-seq dataset were obtained from the MOSCATO–1 clinical trial, whereas RNA-seq data for the deeply sequenced samples, M2594 and M2673, were obtained from the MOSCATO–2 clinical trial; all RNA-seq data were processed as described under “RNA-seq processing and quantification.” LNCaP transcript-per-million (TPM) values were obtained from the Gene Expression Omnibus (GEO; accession GSE128749) using control samples only (*n* = 3)^66^. Protein-coding transcripts were aggregated at the gene level, intersected with genes included in the promoter and gene-body reference (Methods, “Development of APEX: Promoter, gene body, and enhancer reference”) and averaged across control samples.

#### MNase-digested chromatin preparation

Mononucleosomal chromatin was prepared from LNCaP cells following a protocol adapted from Verzi *et al*., *Dev. Cell*, 2010^67^. Briefly, 3 × 10^7^ cells were washed in ice-cold PBS containing protease inhibitors (Sigma-Aldrich, Cat. #11873580001), scraped, and pelleted by centrifugation (2500 × g, 3 min, room temperature). The pellet was resuspended in 900 μL digestion buffer (50 mM Tris-HCl, pH 7.6, 1 mM CaCl_2_, 0.2% NP-40, 5 mM sodium butyrate, complete protease inhibitor cocktail, and 0.5 mM phenylmethylsulfonyl fluoride) and Dounce-homogenized for 1 min. After a 5-min incubation on ice, samples were warmed to 37 °C for 2 min and digested with 3 μL micrococcal nuclease (MNase; 0.2 U/μL, Sigma-Aldrich #N3755) for 7 min at 37 °C. The reaction was stopped by addition of 4.5 μL 0.5 M EDTA (final 2.5 mM). Digested chromatin was briefly sonicated for 30s and then resuspended in dilution buffer (1% NP-40, 0.5% sodium deoxycholate, 0.1% SDS, and complete protease inhibitor cocktail in PBS). Mononucleosomal enrichment (~146 bp) was assessed using the Agilent 4150 TapeStation system and analysis software v3.2 (Agilent Technologies, Inc., Santa Clara, CA). Lysates were centrifuged (14,000 × g, 10 min, 4 °C), and the supernatant containing soluble chromatin was collected and stored at −80 °C until use.

#### Covaris-sheared chromatin preparation

Fixed chromatin was fragmented by ultrasonication to generate random DNA fragments. Briefly, adherent LNCaP cells (1 × 10^7^) were crosslinked by directly adding formaldehyde to the culture medium to a final concentration of 1% and incubating for 10 min at room temperature. Crosslinking was quenched with 125 mM glycine for 5 min at room temperature. Cells were washed twice with ice-cold PBS containing complete protease inhibitors (Sigma-Aldrich, Cat. #11873580001), scraped, and pelleted by centrifugation (625 × g, 4 min, 4 °C). Pellets were resuspended in 1 mL dilution buffer (1% NP-40, 0.5% sodium deoxycholate, 0.1% SDS, and complete protease inhibitors in PBS) and incubated on ice with gentle rocking for 10 min. Chromatin was sheared using the Covaris E220 focused-ultrasonicator (1 mL tubes; peak incident power = 140, duty factor = 5%, burst cycle = 200) for 25 min at 4 °C. Lysates were centrifuged (14,000 × g, 10 min, 4 °C), and the supernatant containing soluble chromatin was collected and stored at −80 °C until use.

### Development of APEX

#### Promoter, gene body, and enhancer reference

Protein-coding genes and their coordinates were obtained from GENCODE comprehensive gene annotation file (GRCh37, release 27) by filtering for entries where type = gene and gene_type = protein_coding. Multimapping gene entries and genes with lengths smaller than 500 bp were excluded. Promoters were defined as a symmetric 3kb window around the transcription start site (TSS), and gene body was defined as the region from the TSS to the transcription termination site (TTS). We found that binning the promoter and gene body into 20 equally sized windows (e.g. a fixed 400 bp promoter bin size and a gene body bin size of $G_{j}/20$, where $G_{j}$ denotes the gene width from TSS to TTS in bp), resulted in significantly better model performance in the training set compared to other bin sizes (**Figures S5G–S5J**).

Enhancer regions were obtained from the GeneHancer database (v5.22; GRCh37)^25^, restricting to high-confidence elements (regulatory_element_type = “Enhancer” and is_elite = 1). Original enhancer coordinates were retained. Enhancers overlapping annotated gene bodies were classified as intragenic (40,342 enhancers across 9,042 genes), whereas those located outside gene bodies were classified as intergenic (26,495 enhancers across 7,564 genes). These promoter, gene body, and enhancer annotations defined the genomic reference used for all downstream feature extraction and modeling in APEX (**Table S4**).

#### Selection of alternate promoters

Promoter activity is tissue- and lineage-specific, and many protein-coding genes use alternative transcription start sites (TSSs) depending on cellular context^68^. Consistent with prior observations of context-specific promoter regulation, we observed that plasma histone modification patterns frequently marked alternative promoters in a cancer-type–dependent manner (**Figure S6**). Across our cohort, 29% of genes exhibited H3K4me3 signal peaks at locations distinct from the GENCODE v27–annotated TSS, and 64% of these alternative promoter assignments varied between the 15 evaluated samples (**Table S4**).

To account for this heterogeneity, candidate TSSs were defined using the proActiv R package (v1.1.18) with the GENCODE GRCh37 GTF file (release 27) as input. Promoter coordinates were derived from the resulting PromoterAnnotation object, and the union of proActiv-defined alternative promoters and GENCODE v27 TSSs was used as the candidate TSS set for each gene (*n* = 66,976 TSSs across 18,493 protein-coding genes; **Table S4**). For each sample, fragment coverage was quantified within ±500 bp of each candidate TSS, and the TSS with maximal H3K4me3 coverage was selected as the optimal promoter. Promoter (TSS ±3 kb) and gene-body (TSS to transcription termination site) coordinates were then updated accordingly. Genes for which TSS reassignment resulted in a residual gene body shorter than 500 bp were excluded from downstream analyses to ensure robust feature estimation.

Dynamic TSS reassignment significantly improved gene-level Spearman rank correlations between plasma-derived features and matched tumor RNA-seq, with the largest gains observed for genes in which the selected TSS deviated by more than 10 kb from the default GENCODE annotation (**Figure S6**). Improvements were most pronounced for narrow histone marks such as H3K4me3 and H3K27ac, whereas broad marks such as H3K36me3 were less affected (**Figure S6**).

Furthermore, because sample-specific TSS selection relies on H3K4me3 signal, we evaluated its performance at low tumor fractions using the experimental SCLC plasma dilution series (see “Evaluation of APEX and cfChIP-seq limit of SCLC detection through serial dilutions”). At each dilution, APEX was applied using either dynamically selected sample-specific TSSs or fixed GENCODE v27 TSSs, and performance was assessed at both the 100-gene SCLC signature and individual-gene levels. Although overall differences between the two approaches were not statistically significant, SCLC detection was reduced at tumor fractions ≤3.125%, raising concern that dynamic TSS selection may increasingly reflect hematopoietic rather than tumor-derived chromatin at very low tumor fractions (**Figure S7**). Accordingly, the APEX software allows users to supply custom promoter annotations. Users may consider disabling dynamic TSS selection when estimated tumor fraction is ≤3.125%, although this threshold is not enforced.

#### Feature Extraction

To develop APEX (Associating Plasma Epigenomics with eXpression), we extracted coverage-, size-, and sequence-based features across 18,342 protein-coding genes (Figures 1B and C). Features were derived from cfDNA co-immunoprecipitated with circulating histones marked by H3K4me3, H3K27ac, or H3K36me3, and across promoter, gene body, intergenic enhancer, and intragenic enhancer regions as defined in “Development of APEX: Promoter, gene body, and enhancer reference” (Figure 1B, **Table S4**). The rationale and definitions for each feature are described below. Notably, for intergenic and intragenic enhancer regions, the reported metric for each gene was calculated as the weighted average across all of that gene’s associated enhancer peaks, as annotated in the GeneHancer database^25^.

##### Coverage per bin.

The total signal from active histone marks (H3K4me3, H3K27ac, and H3K36me3) and its distribution across promoters, enhancers (intergenic and intragenic), and gene bodies has been associated with gene expression^16^. Therefore, we quantified coverage for each bin i and gene j in region p as follows:

$${\text{Coverage}}_{i,j,p}={\text{log}}_{2}\left(\frac{\frac{C_{i,j,p}}{B_{i,j,p}\times 10^{-3}}}{\sum_{n=1}^{N}\frac{C_{i,n,p}}{B_{i,n,p}\times 10^{-3}}}\times 10^{6}+0.01\right),$$

where counts $\left(C_{i,j,p}\right)$ represent unique fragments overlapping the bin, calculated as the number of unique fragments overlapping that bin using the findOverlaps function in the GenomicRanges R package (v1.56.2)^57^.

To account for length-related biases, counts were normalized by the bin width in kilobases, or $\left(B_{i,j,p}\right)$, which varied according to the number of bins, the region evaluated, and the gene considered. Specifically, promoter regions were defined as a fixed 6 kb window centered on the transcription start site (TSS; unless otherwise specified); gene body regions spanned the distance from the TSS to the transcription termination site (TTS), with TSS coordinates defined by Gencode v27 or adjusted to the optimal TSS as described under “Development of APEX: Selection of alternate promoter”; and intergenic or intragenic enhancer regions were defined by the weighted average (Methods, “Development of APEX: Promoter, gene body, and enhancer reference”) of widths corresponding to each gene’s associated enhancer peaks as annotated in the GeneHancer database^25^.

Bin-width–normalized counts were scaled by the total number of fragments (in millions) aligning to the same bin position across all genes in that sample to account for bin-specific and sample-level differences in sequencing depth. The resulting values were log_2_-transformed with a small pseudocount (0.01), chosen to introduce minimal bias relative to larger pseudocounts while providing slightly improved performance in the training cohort (**Figure S5D**).

##### Maximum coverage per region.

We hypothesized that the maximal signal intensity of active histone marks, such as H3K4me3, H3K27ac, and H3K36me3, may correlate with gene expression activity. For each gene j and region p, the bin i with the highest coverage among the $n_{j,p}$ bins was identified and included as a model feature:

$${\text{MaxCoverage}}_{j,p}=\underset{i=1,\ldots ,n_{j,p}}{\text{max}}\left({\text{Coverage}}_{i,j,p}\right).$$

##### Frequency of differently sized fragments per region.

The distribution of cfDNA fragment lengths can reflect patterns of transcription factor– and nucleosome–protected DNA^69^. Fragment length frequencies have also been associated with locus-specific methylation and histone mark activity^70,71^. In our study, we identified fragment size windows that were most strongly associated with gene expression in the training cohort (Figures 2A, 2E, and 2F). Based on these findings, we defined five fragment size windows: 20–80 bp, 160–200 bp, 280–320 bp, 400–440 bp, and all remaining fragments shorter than 500 bp.

For each gene j and region p, the frequency of fragments within each window w was calculated as a feature in the model:

$${\text{FragFreq}}_{j,p,w}=\frac{N_{j,p,w}}{\sum_{w}N_{j,p,w}},$$

where $N_{j,p,w}$ is the number of fragments of size window w in region p of gene j, and the denominator sums the counts of fragments across all windows w for that same gene j and region p. This normalization produces a relative frequency for each fragment size window, allowing comparisons across genes and regions while accounting for differences in total fragment counts.

##### Mean and variance of fragment length per bin.

Following the rationale described in “Frequency of differently sized fragments per region”, we also computed the mean and variance of fragment lengths for each bin i and gene j in region p.

$${\text{MeanFL}}_{i,j,p}=\frac{1}{N_{i,j,p}}\sum_{f=1}^{N_{i,j,p}}L_{f},$$

$${\text{VarFL}}_{i,j,p}=\frac{1}{N_{i,j,p}}\sum_{f=1}^{N_{i,j,p}}{\left(L_{f}-{\text{MeanFL}}_{i,j,p}\right)}^{2},$$

where $L_{f}$ is the length of fragment f overlapping bin i of gene j in region p, and $N_{i,j,p}$ is the total number of fragments overlapping that bin.

##### Shannon entropy of fragment length per bin.

Previous studies have associated fragment length diversity near the TSS with gene expression^9^. However, the relationship between the diversity of nucleosome-immunoprecipitated fragments and gene expression has not been explicitly assessed. To evaluate this, we calculated fragment length frequencies across a range of 20–500 bp, as follows:

$${\overset{ˆ}{p}}_{i,j,p,l}=\frac{n_{i,j,p,l}}{n_{i,j,p}}\text{for l}\in [20,500]$$

where $n_{i,j,p,l}$ denotes the number of fragments of length l in bin i of gene j and region p, and $n_{i,j,p}$ represents the total number of fragments in that bin. We then computed the Shannon entropy of these distributions within each bin i in gene j and region p:

$$H_{i,j,p}=-\sum_{l=20}^{500}{\overset{ˆ}{p}}_{i,j,p,l}\cdot {log}_{2}\left({\overset{ˆ}{p}}_{i,j,p,l}\right).$$

Entropy values were normalized by dividing by $log_{2}(L)$, where L is the total number of fragment length bins (481 for the 20–500 bp range), to normalize values between 0 and 1.

$$H_{i,j,p}^{\text{norm}}=\frac{H_{i,j,p}}{{log}_{2}(L)}.$$

Lower entropy values indicate reduced diversity or a highly skewed fragment length distribution, while higher values reflect greater diversity or a more uniform distribution.

##### Frequency of 4-mer end motifs per region.

End-motif sequence biases have been associated with specific histone modifications, nucleosome positions, and tissue-specific enzymes^12,13^. To avoid potential distortions of 3′ end motifs caused by end-repair and A-tailing during double-stranded DNA library preparation, we analyzed motifs at the 5′ ends of fragments. Using bedtools getfasta (v2.30.0)^56^, we extracted 4-mer nucleotide motifs from the 5′ end of both the positive and negative fragment strands, yielding two end-motifs per fragment. For each gene j and 4-mer motif m in region p, the motif frequency was calculated as a relative fraction:

$${\text{MotifFreq}}_{j,p,m}=\frac{N_{j,p,m}}{\Sigma_{m}N_{j,p,m}},$$

where $N_{j,p,m}$ is the count of motif m in region p of gene j, and the denominator sums the counts of all 256 possible 4-mer motifs within the same gene j and region p. This normalization produces a relative frequency for each motif, allowing comparisons across genes and regions while accounting for differences in total fragment counts.

##### Shannon entropy of 4-mer end motifs per region.

Several studies have suggested that the distribution of end-motif sequence biases can be associated with locus-specific epigenomic activity^12,13^. To evaluate this, we computed a motif diversity score (MDS), defined as the Shannon entropy of 4-mer frequencies for each gene j and region p across all L=256 possible combinations of ‘A’, ‘C’, ‘T’, and ‘G’. For each motif m, the relative frequency in region p of gene j was calculated as:

$${\overset{ˆ}{p}}_{j,p,m}=\frac{n_{j,p,m}}{n_{j,p}},$$

where $n_{j,p,m}$ is the number of fragments with motif m in region p of gene j, and $n_{j,p}$ is the total number of fragments in that region. The Shannon entropy of 4-mer end motifs $\left({\text{MDS}}_{j,p}\right)$ for gene j and region p was then computed as:

$${\text{MDS}}_{j,p}=-\sum_{m=1}^{L}{\overset{ˆ}{p}}_{j,p,m}{log}_{2}\left({\overset{ˆ}{p}}_{j,p,m}\right).$$

To facilitate comparisons across genes and regions, MDS values were normalized to the theoretical maximum entropy:

$${\text{MDS}}_{j,p}^{\text{norm}}=\frac{{\text{MDS}}_{j,p}}{{\text{log}}_{2}(L)},$$

yielding values between 0 and 1. Lower MDS values indicate reduced sequence diversity or a strong preference for specific 4-mer end motifs, whereas higher values reflect greater diversity or a more uniform motif distribution.

##### Average fragment GC content per bin.

The GC content of DNA fragments contributes substantially influences coverage variability through both technical factors (e.g., PCR amplification bias, library preparation methods) and biological factors (e.g., promoter CpG density, transcription factor binding sites [TFBSs]). Distinguishing technical artifacts from true biological signals in ChIP-seq is particularly challenging because immunoprecipitated DNA is often enriched for GC-rich regions, especially near promoters and TFBSs. Although GC bias correction methods have been proposed for transcription factor ChIP-seq^72^, standardized approaches for histone ChIP-seq are lacking.

To capture potential GC-related effects, we incorporated the mean GC content of fragments within each bin as a feature. GC content was computed using bedtools nuc (v2.30.0)^56^ as the proportion of guanine and cytosine bases per fragment. For each bin i and gene j in region p, the mean GC content $\left({\bar{GC}}_{i,j,p}\right)$ was calculated as:

$${\bar{GC}}_{i,j,p}=\frac{1}{n_{i,j,p}}\sum_{f=1}^{n_{i,j,p}}\lambda_{f},$$

where $n_{i,j,p}$ is the number of fragments in that bin, and $\lambda_{f}$ is the GC fraction of fragment f, computed from the number of guanine $\left(G_{f}\right)$ and cytosine $\left(C_{f}\right)$ bases divided by the total fragment length $\left(L_{f}\right)$ :

$$\lambda_{f}=\frac{G_{f}+C_{f}}{L_{f}}.$$

All cfChIP-seq features were compiled at the gene level to generate a unified feature matrix. For the initial analysis of the training cohort and *in silico* screen (Figs. 1–2), each annotated region was evenly tiled into five bins, yielding 3,516 parameters per sample. Features included bin-level coverage, maximum coverage per region, fragment-length statistics (Shannon entropy, mean, and variance), 4-mer end-motif entropy and frequencies, and GC content, computed for each histone modification (H3K4me3, H3K27ac, and H3K36me3). Following feature optimization (Methods, “Development of APEX: Feature and model optimization”), promoter and gene-body regions were divided into 20 bins, while intragenic and intergenic enhancer regions remained untiled (one bin each), resulting in 3,912 parameters.

###### Feature normalization

To normalize feature distributions across samples and reduce the impact of extreme values, we compared empirical cumulative distribution function (ECDF) normalization with robust median/IQR scaling, z-score normalization, and no normalization (**Figure S5C**). All approaches were evaluated using the same feature matrix, model architecture, and cross-validation framework, with model performance summarized by cross-validated RMSE. ECDF, robust scaling, and z-score normalization each improved performance relative to unnormalized features, supporting the use of feature normalization before model training. ECDF normalization was used in APEX because it achieved the lowest median RMSE and provided a nonparametric transformation that is less sensitive to outliers.

For each feature vector, the ECDF was calculated using all non-missing, non-zero observations, with zeros excluded to avoid distortion of the lower tail. For each valid observation $x_{i}$, the transformed value $F\left(x_{i}\right)$ was calculated as:

$$F\left(x_{i}\right)=\frac{1}{n}\sum_{j=1}^{n}1\left(x_{j}\leq x_{i}\right),$$

where n represents the number of valid observations, and 1(·) is the indicator function, which equals 1 if the condition $x_{j}\leq x_{i}$ is met and 0 otherwise. Each observation was replaced by its empirical percentile, whereas zero and missing values were excluded from the rank estimation and retained as NA.

#### Regression model selection

To identify the optimal statistical framework for predicting gene expression from cfChIP-seq features, we trained and compared multiple regression models in *R* (v4.4.2). In total, we evaluated seven regression frameworks: ordinary least squares (OLS), ridge, lasso, and elastic-net regression (*glmnet*, v4.1–8)^73^; partial least squares regression (*pls*, v2.8–1); random forest regression (*ranger*, v0.16.0); and gradient boosting regression (*xgboost*, v3.2.1)^74^. The input matrix comprised ECDF-normalized chromatin features (17,941 genes × 3,516 features) across 15 training samples (269,115 total gene–sample observations). Missing values for individual features were imputed with their median across all observations. The outcome variable for each gene–sample observation was log_2_ (TPM + 1) gene expression.

Linear regression models were fit using *glmnet* with α=0 (ridge), α=1 (lasso), or α=0.5 (elastic-net). Each model was trained using 5-fold cross-validation, minimizing mean squared error (MSE), and predictive performance was summarized by the root-mean-squared error (RMSE) on held-out data. OLS regression was implemented as ridge regression with a minimal penalty $\left(\lambda =10^{-8}\right)$. Partial least squares regression was fit with up to 30 components using segment-wise cross-validation. Tree-based models were trained with default parameters: random forests (*ranger*) with 500 trees, and gradient boosting (*xgboost*) with 500 boosting rounds and early stopping after 20 rounds without improvement.

All models were evaluated using stratified 5-fold cross-validation, repeated 10 times to ensure stability. Performance was assessed by the RMSE on held-out data. The optimal model was defined as the one yielding the lowest mean RMSE, with significance determined by a paired Wilcoxon signed-rank test (*P* < 0.05) against other models.

#### Feature and model optimization

Feature and model parameters were optimized in a two-stage grid search strategy, allowing genomic features to be fixed before tuning model parameters (**Figure S5E–S5O**). In the first stage, parameters defining the resolution of promoter, gene body, intragenic, and intergenic regions were tuned to maximize predictive performance. Candidate feature configurations were drawn from a grid specifying the number of promoter, gene body, intragenic enhancer, and intergenic enhancer tiles (1, 5, 20, or 40 each), and, for promoters, the upstream and downstream window sizes relative to the TSS (500, 1,000, 3,000, or 5,000 bp). From all possible combinations, 300 were randomly sampled for evaluation.

Each parameter set was evaluated using an XGBoost regression model in R (v.3.2.1; default parameters: η=0.3, max_depth = 6, γ=0, colsample_bytree = 1, subsample = 1, nrounds = 500, early_stopping_rounds = 20, tree_method = “hist”, objective = “reg:squarederror”, and evaluation metric = “rmse”). Model performance was assessed by five-fold cross-validation, where 15 samples were randomly partitioned into five folds. To reduce sampling bias and yield stable performance estimates, cross-validation was repeated ten times with distinct random seeds defined as *set.seed* = 42 + *i* for the *i*-th repeat. For each configuration, the mean RMSE across the ten repeats was recorded, and parameter importance was assessed using the Wilcoxon rank-sum test to identify the optimal feature set minimizing RMSE (**Figure S5E–S5J**).

In the second stage, XGBoost hyperparameters were optimized using the best-performing feature configuration (20 promoter tiles, 20 gene body tiles, one intragenic tile, one intergenic tile, and 3,000 bp upstream and downstream windows). A grid of 243 hyperparameter combinations was evaluated, varying learning rate (η∈{0.05,0.1,0.2}), maximum tree depth (max_depth ∈ {3, 5, 7}), minimum loss reduction (γ∈{0,0.1,1}), column subsampling rate (colsample_bytree ∈ {0.5, 0.7, 1.0}), and row subsampling rate (subsample ∈ {0.6, 0.8, 1.0}). The same 5-fold, ten-repeat cross-validation strategy was used to compute the mean RMSE for each combination, and the parameter set yielding the lowest mean RMSE was selected for final model training. Where multiple settings yielded comparable performance, parameters favoring greater regularization were selected. For example, a column subsampling rate of 0.7 was chosen despite similar RMSE at 1.0 to reduce overfitting among correlated features, and γ=1 was selected as a conservative setting given similar performance across the tested range (**Figure S5K–S5O**).

#### Model training and selection

Final XGBoost regression models were trained on the complete ECDF-normalized feature matrix comprising 3,912 features across 275,130 observations (18,342 genes × 15 samples), with log_2_ (TPM + 1) RNA-seq expression as the target variable. Training was performed using the xgb.train function^74^ in R with hyperparameters identified during model optimization. Of the 3,912 input features, 3,573 contributed predictive value and were retained in the full model.

To balance predictive performance and computational efficiency, we developed four APEX variants. APEX–full incorporated all 3,573 features. A refined multi-mark model incorporated only promoter and gene-body features from H3K4me3 and H3K36me3, reducing the feature set from 3,573 to 1,532 features. Single-mark variants (766 features each) were also evaluated for parsimony, including APEX–K4 and APEX–K36.

Runtime performance was evaluated on the training cohort using an Apple M3 Max MacBook Pro (16-core CPU, 64 GB RAM) with optimized XGBoost parameters (maximum depth = 7, minimum loss reduction = 1.0, feature fraction = 0.7, learning rate = 0.10, sample fraction = 1.0). The full model required a median of 10.5 minutes per sample, whereas the refined H3K4me3 + H3K36me3 model required 6.5 minutes per sample without loss of predictive accuracy (**Figure S8G**). Single-mark variants further reduced runtime (median 2.5 minutes for H3K4me3 and 4.2 minutes for H3K36me3) but at the cost of reduced predictive performance (**Figure S8F and S8G**).

Based on predictive accuracy, feature complementarity, non-redundancy, and runtime efficiency, we selected the refined APEX model incorporating H3K4me3 and H3K36me3 promoter and gene-body features as the preferred framework for downstream analyses (**Figure S8H**).

### Robustness of APEX model performance to the number of gene–sample pairs

To assess the robustness of model performance to the number of gene–sample observations, we evaluated how training sample size affects model performance. Briefly, we trained models with different gene–sample pairs in the training, using the optimized feature matrix (n=1,532 features; parameters: 20 promoter and gene body tiles for H3K4me3 and H3K36me3 with promoter regions defined as ±3 kb around the TSS) and optimized XGBoost configuration (η=0.1, max_depth = 7, γ=1, colsample_bytree = 0.7, subsample = 1, tree_method = “hist”).

Model performance was evaluated using leave-one-out cross-validation (LOOCV), where models were trained on a downsampled (e.g. 200,000, 150,000, 100,000, 50,000, 30,000, 20,000, 10,000, 5,000, 1,000, 100, and 10 observations) or full feature matrix (e.g. 256,788 gene–sample observations for 14 samples) and tested on all genes in a held-out sample (18,342 genes). Predictive accuracy was quantified as the RMSE on the held-out sample.

The minimum number of gene–sample pairs required to maintain robust performance was defined as the smallest subset size whose RMSE was not significantly higher than that of the full model, as determined by an unpaired Wilcoxon rank-sum test (*P* ≥ 0.05). Robust performance was preserved down to ~30,000 gene–sample pairs (**Figure S12A**).

### Robustness of model performance to noise in tumor RNA-seq labels

Tumor gene expression measurements can vary substantially due to differences in tumor purity and spatial heterogeneity. Moreover, multiple metastatic sites can contribute to signal in plasma cfDNA. To assess the stability of model performance under such variability, we evaluated robustness to label perturbation.

The same optimized feature matrix and hyperparameters were used, with leave-one-out cross-validation (LOOCV) as described above.

Gaussian noise was added to tumor RNA-seq labels in the training folds only (256,788 gene–sample observations across 14 samples per iteration), scaled to a specified fraction of the global standard deviation of expression values (0–10×), and truncated at zero to avoid negative values. Scaling noise to the global rather than gene-specific standard deviation ensured that perturbation magnitude was independent of intrinsic expression variability, which is correlated with biological signal strength. For each noise level (0, 0.2, 0.4, 0.6, 0.8, 1, 2, 3, 5, and 10×), models were trained on label-perturbed data and evaluated on the unperturbed held-out sample (18,342 genes). Model performance was summarized as the RMSE between predicted and observed expression.

The highest noise level at which RMSE remained statistically indistinguishable from the noise-free baseline (unpaired Wilcoxon rank-sum test, *P* ≥ 0.05) was defined as the robustness threshold. The model remained stable up to a perturbation amplitude of approximately 0.6× the global standard deviation, indicating resilience to moderate variability in tumor RNA-seq measurements.

### Evaluation of APEX generalizability across cancer types

To assess the robustness of APEX across diverse tumor contexts, we evaluated model performance using two complementary cross-cancer generalizability analyses. First, within the training cohort, we performed a leave-one-cancer-out (LOCO) procedure. The 15 training samples were grouped into seven tumor types, and for each iteration, APEX was trained on six cancer groups and evaluated on all gene–sample pairs from the excluded cancer type. This analysis quantified the extent to which models trained on a subset of cancers could predict gene expression programs in an unseen malignancy. LOCO performance was compared directly with leave-one-sample-out cross-validation (LOOCV), in which the model was trained on 14 samples and evaluated on the held-out sample. Across all iterations, no significant difference in RMSE was observed between LOCO and LOOCV, indicating that APEX performance was stable even when entire cancer types were omitted from training (**Figure S12D**).

To further evaluate external generalizability, we assessed APEX in an independent cohort from the MOSCATO–2 clinical trial, which comprised 34 samples spanning 18 cancer types, 12 of which were not represented in the training cohort (see “MOSCATO clinical cohort”). For each sample, Spearman correlation was computed between APEX-predicted gene expression and matched bulk tumor RNA-seq. Performance in MOSCATO–2 was comparable to that observed in the training cohort, despite differences in cohort composition, sample handling, and cancer representation (Figures 3F and **S12F**). Together, these analyses demonstrate that APEX maintains predictive accuracy across a broad range of tumor types and generalizes to malignancies not encountered during model development.

### Feature contribution and importance analysis

To assess the contribution of individual feature classes and the relative importance of specific features, we performed systematic feature-ablation and permutation-based importance analyses. For each ablation, models were retrained after excluding predefined feature groups, including genomic regions (promoter, gene body, intergenic, and intragenic), histone marks (H3K4me3, H3K27ac, and H3K36me3), or cfDNA-derived feature categories (coverage, GC content, fragment length, and 4-mer end-motif metrics), using otherwise identical training and cross-validation procedures. Model performance was evaluated by leave-one-out cross-validation and summarized as the root-mean-squared error (RMSE) between predicted and observed expression. Histone-mark ablation was additionally performed in the independent validation cohort of 34 matched plasma–tumor pairs. Changes in RMSE after removal of each histone mark were evaluated across the full cohort and stratified by cancer type. Statistical significance of differences in RMSE was assessed using paired Wilcoxon signed-rank or Kruskal–Wallis tests, as appropriate.

To quantify the relative influence of individual chromatin features, we applied a model-agnostic permutation-based importance framework (DALEX v2.5.3, R)^75^. For each feature, its values were randomly permuted across samples in the final XGBoost model (trained on H3K4me3 and H3K36me3 promoter and gene body features), and the resulting degradation in model accuracy was used to estimate importance. The median loss in predictive performance across all permutations provided a stable measure of each feature’s contribution, enabling unbiased ranking of the most informative chromatin determinants of gene expression.

### Reproducibility of APEX feature importance across independent cohorts

To evaluate the reproducibility of the chromatin-feature architecture learned by APEX, an independent XGBoost regression model (R package xgboost, v3.2.1.1) was trained de novo in the original validation cohort of 34 patients spanning 18 cancer types, using 623,594 gene–sample observations (34 patients × 18,341 genes), the same prespecified set of 1,532 H3K4me3- and H3K36me3-derived promoter and gene-body features, and the same hyperparameters used for the primary APEX model (Methods, “Development of APEX”). This model was used solely to assess feature stability and not to estimate validation performance. Feature importance was quantified by gain using xgb.importance, and concordance with the original 15-patient training model was assessed using Spearman and Pearson correlations across all features, together with the overlap among the ten highest-ranked features and their cumulative contribution to total feature gain.

### Gene-level determinants of prediction performance

To identify gene-level characteristics associated with APEX prediction performance, we calculated the root-mean-squared error (RMSE) between APEX-inferred and measured tumor RNA-seq expression for each gene across the independent validation cohort of 34 matched plasma–tumor pairs. For visualization, genes were grouped according to baseline tumor expression, housekeeping-gene status, tissue specificity, whole-blood association, gene length, and promoter CpG-island status, and the distributions of per-gene RMSE were compared across these categories.

Baseline tumor expression was defined as the mean measured RNA-seq expression across the 34 tumors, and genes were divided into tertiles representing low, intermediate, and high expression. Gene length was calculated from GENCODE gene-body coordinates (GRCh37, release 27) and similarly divided into tertiles representing short, intermediate, and long genes. Housekeeping genes were defined using a published set of 3,804 genes exhibiting uniform expression across human tissues^76^. Tissue-specific genes were defined as the union of genes assigned to normal-tissue-specific GTEx signatures, whereas whole-blood-associated genes were defined using the GTEx whole-blood signature (see “Defining cancer- and normal-subtype-specific gene signatures”). These annotations were treated as binary variables. Promoter CpG-island status was assigned according to whether a fixed interval extending 1 kb upstream and downstream of the annotated TSS overlapped a UCSC CpG-island annotation for GRCh37/hg19.

To evaluate the independent association of each gene characteristic with prediction error, we fitted a multivariable linear regression model using the R stats package (v4.4.2), with the natural logarithm of per-gene RMSE as the outcome. Covariates included mean tumor expression, housekeeping-gene status, tissue-specific status, whole-blood association, log10-transformed gene length, and promoter CpG-island status. Continuous predictors were centered and scaled to a mean of zero and standard deviation of one before model fitting. Model coefficients and 95% confidence intervals were transformed back to the original RMSE scale and reported as the adjusted percentage change in per-gene RMSE.

### Evaluation of gene class-specific models

To assess whether training models within biologically defined gene classes improved prediction accuracy, genes were stratified by baseline tumor expression, gene length, housekeeping-gene status, transcription-factor identity, and tissue specificity. Baseline expression, gene length, housekeeping status, and tissue specificity were defined as described in the “Gene-level determinants of prediction performance“ section. Transcription factors were defined using the University of Toronto Human Transcription Factors database (v1.01), comprising 1,639 transcription-factor genes (accessed June 26, 2026).

Separate XGBoost regression models were trained within each gene category using the same 1,532 H3K4me3- and H3K36me3-derived features and the same hyperparameters used for the global APEX model. Category-specific and global models were evaluated on identical sets of genes and held-out patients using patient-level leave-one-out cross-validation. For each gene, root-mean-squared error was calculated across held-out patients, and prediction-error distributions were compared descriptively between the global model and the corresponding category-specific model. Individual gene-specific models were not trained because the number of patient-level observations available for each gene was insufficient for reliable model fitting.

### Benchmarking against existing fragmentomic and cfChIP-seq methods

To benchmark APEX against established fragmentomic and cfChIP-seq-based gene-expression inference methods, we selected five samples from the independent validation cohort spanning a range of tumor types, tumor fractions, and pre-library cfDNA inputs: M2187 (uterine; 19.55 ng cfDNA; ctDNA 0.45; 92× depth), M2240 (esophageal; 20 ng; ctDNA 0.37; 93×), M2246 (lung; 100 ng; ctDNA 0.77; 101×), M2331 (cholangiocarcinoma; 16.51 ng; ctDNA 0.05; 114×), and M2692 (colorectal; 32 ng; ctDNA 0.20; 117×). Sequencing depths (90–120×) were selected to match ranges commonly used in prior fragmentomic studies. For each sample, 1 mL of plasma was independently profiled by cfChIP-seq for H3K4me3, H3K27ac, and H3K36me3 using the workflow described above.

APEX predictions were generated as described above. H3K4me3 and H3K27ac promoter signals were quantified within ±3 kb of GENCODE v27 transcription start sites and normalized to DNase I hypersensitive site (DHS) coverage to control for global chromatin accessibility^17,77^. H3K36me3 gene-body coverage was computed from annotated TSS to TTS, normalized by gene width and total fragment counts to account for sequencing depth and fragment-length distributions.

To compare with fragmentomic approaches, we used the publicly available implementation and recommended workflow from the EPIC-seq study (EPICSeqCode.v0.tar; https://epicseq.stanford.edu)^9^. Briefly, paired-end reads were trimmed using fastp v0.24.0^78^, aligned to hg19 using BWA-MEM v0.7.17 with extended insert-size modeling (-I 250,200,2000,10)^55^, and processed with samtools v1.15.1 to retain uniquely mapped fragments (MAPQ ≥ 1)^79^. Reads were sorted, deduplicated with Picard MarkDuplicates v2.6.0 (RRID:SCR_006525), and further filtered using samtools view -h -q 30 -F 3084, followed by restriction to canonical properly paired configurations (BAM flags 81, 83, 97, 99, 145, 147, 161, 163)^79^.

Fragmentomic features were computed using the publicly released EPIC-seq code from https://epicseq.stanford.edu^9^, including EPIC-seq-inferred expression, promoter fragmentation entropy (PFE), Gini index, orientation-aware fragmentation, Shannon index of fragment lengths, nucleosome depletion ratio (NDR), and GC content. One modification was applied to the PFE normalization step, informed by with the original developers: the gamma-distribution shape parameter used in the pgamma (R stats v4.4.2) transformation was changed from 0.5 to 0.25 to better reflect entropy distributions in deeply sequenced cfDNA libraries and to avoid compression of values toward 1. All other components of the EPIC-seq pipeline were executed exactly as provided in the original code.

Because EPIC-seq uses Ensembl v75 TSS annotations, gene symbols were harmonized to GENCODE v27 using HGNChelper v0.8.15, yielding 17,348 shared genes for comparison^65^.

Predictive performance was assessed by computing gene-level Spearman correlations between each method’s inferred expression and matched tumor bulk RNA-seq (log_2_ (TPM+1)), using pairwise complete observations. Recovery of cancer-type-specific transcriptional programs was evaluated using the top 10 tumor-specific genes defined from matched bulk RNA-seq (see “Defining cancer- and normal-subtype-specific gene signatures”) and mean predicted expression of these gene sets was visualized as a 5×5 heatmap.

### Comparison of cfChIP-seq and deep whole-genome sequencing inputs

To determine whether regional coverage and fragmentomic features from unenriched cfDNA could reproduce APEX performance, we analyzed five patients with matched deep whole-genome sequencing (WGS), H3K4me3 and H3K36me3 cfChIP-seq, and tumor RNA-seq data (Methods, “Benchmarking against existing fragmentomic and cfChIP-seq methods”). For each WGS sample, we extracted the same 1,532 promoter- and gene-body-derived coverage and fragmentomic features used for cfChIP-seq and generated predictions with the fixed cfChIP-seq-trained APEX model, using identical feature definitions, preprocessing, and learned weights. Thus, this analysis assessed the information recoverable from unenriched cfDNA within the APEX framework rather than a model independently optimized for WGS. Performance was measured across 18,333 genes per patient as the Spearman correlation between inferred and matched tumor RNA-seq expression and compared between paired WGS and cfChIP-seq inputs using a two-sided paired t-test. Sequencing input was summarized by the number of fragment observations across the H3K4me3- and H3K36me3-derived feature spaces, with cfChIP-seq counts representing the combined number of unique fragments from both marks.

### Evaluation of APEX and cfChIP-seq limit of SCLC detection through serial dilutions

#### Serial dilution design and cfChIP-seq profiling

To evaluate whether APEX improves sensitivity for detecting tumor-derived chromatin signals beyond individual histone features, we performed a serial-dilution experiment using plasma from three patients with small-cell lung cancer (SCLC; tumor fractions 0.31, 0.41, and 0.55) and three non-cancer donors. Plasma cfDNA was extracted as described above under “Cell-free DNA isolation and low-pass whole genome sequencing (LP-WGS)”, and each SCLC plasma sample was diluted to match the cfDNA concentration of its paired non-cancer donor using dilution buffer (1% NP-40, 0.5% sodium deoxycholate, 0.1% SDS, protease inhibitor cocktail in PBS). To confirm dilution linearity, each sample was spiked with 192 pg of recombinant nucleosomes from the SNAP-ChIP^®^ K-AcylStat^™^ Panel (EpiCypher #19–3100), including ~8.3 pg of an H3K27ac nucleosome. Spike-in recovery was quantified following H3K27ac immunoprecipitation using qPCR on a LightCycler 480 with PowerUp^™^ SYBR^™^ Green Master Mix (Thermo Fisher Scientific, A25742; software version 1.5.1.62-SP3) and a universal forward primer (5′-CGTCGAACGCGCGATAT-3′) paired with an H3K27ac-specific reverse primer (5′-CGTATACGCGCGACA-3′).

For each SCLC–non-cancer pair, SCLC plasma was mixed with equimolar non-cancer plasma to generate a dilution series corresponding to the original tumor fraction multiplied by 1, 1/2, 1/4, 1/8, 1/16, 1/32, and 1/64 and a 0%-tumor (non-cancer-only) reference. Libraries were generated for all mixtures using the cfChIP-seq workflow described above under “Cell-free chromatin immunoprecipitation sequencing (cfChIP-seq)” and processed to obtain histone-mark coverage profiles and APEX-inferred gene-expression values. Although recombinant H3K27ac spike-in quantification demonstrated appropriate immunoprecipitation at all dilutions, endogenous H3K27ac yielded low total fragment numbers and enrichment scores and was therefore excluded from these analyses.

#### Signature-level limit of detection analysis

To estimate the analytical limit of detection, we quantified recovery of a SCLC-specific gene expression signature across the dilution series, using a previously published approach^14^. For each mixture, signal was computed using the top 100 genes from a predefined SCLC-specific geneset and normalized to a 500-gene WBC-specific background signature (*n* = 443 overlapping genes; see “Defining cancer- and normal-subtype-specific gene signatures”; **Table S8**).

For each sample, APEX or histone coverage values were calculated for each SCLC gene and normalized for background WBC-derived signal by dividing by the mean signal across WBC-signature genes within the same sample. The resulting 100 normalized values per dilution condition were compared with the 0% non-cancer reference using a two-sided *t*-test, and the corresponding *t*-statistic was reported. Three biological replicates were included per dilution, except for the 6.25% condition, which included two replicates. For each plasma metric, the relationship between dilution fraction (*x*-axis) and mean *t*-statistic (*y*-axis) was modeled using a logistic growth function implemented in GraphPad Prism (v10.4.1):

$$Y=\frac{Y_{\text{max}}\cdot Y_{0}}{\left(Y_{\text{max}}-Y_{0}\right)\cdot e^{-kx}+Y_{0}},$$

where Y is the predicted mean *t*-statistic at dilution fraction $x,Y_{\text{max}}$ is the asymptotic maximum *t*-statistic, $Y_{0}$ is the *t*-statistic at zero tumor fraction, and k is the growth rate constant. A *t*-statistic threshold of 2 (approximately corresponding to a two-sided *P* = 0.05) was selected as the detection cutoff.

#### Single-gene-level limit of detection analysis

We also evaluated the analytical limit of single-gene detection across the top 100 genes from a predefined set of SCLC-associated genes, including *CHGA*, a lineage-specific marker for SCLC^27^, and *DLL3*, a clinically actionable target in SCLC^28^. For each dilution condition, gene-specific enrichment was calculated as signal relative to the non-cancer baseline using APEX-inferred expression, H3K4me3 promoter coverage, or H3K36me3 gene-body coverage.

To assess statistical significance, a null background distribution was generated by computing the same enrichment metric for all WBC-specific genes (*n* = 443). To avoid divisions by zero, a pseudocount of 1 was added to both the numerator and denominator. An empirical *P*-value were determined for each gene and dilution as the fraction of WBC-signature genes with enrichment equal to or exceeding that observed for the gene of interest. A gene was considered detected at a given dilution if enrichment relative to the WBC background exceed the WBC distribution and reached an empirical *P* < 0.05. For each gene, the limit of detection was defined as the lowest tumor fraction at which this criterion was met.

### Heterogeneity-aware in silico dilution analysis

To assess whether APEX retained patient- and biopsy-specific transcriptional programs during dilution, we selected two patients with non-small cell lung cancer from the independent validation cohort who had matched plasma cfChIP-seq and tumor RNA-seq obtained from different biopsy sites. Patient M2246 had a primary lung tumor biopsy, whereas patient M2180 had a lymph-node metastasis. For each patient, a tumor-specific transcriptional signature was defined as the 100 genes with the largest difference between expression in that patient’s tumor and the mean expression across all other tumors in the validation cohort. The two signatures exhibited approximately 5% gene overlap.

Serial in silico dilutions were generated independently for H3K4me3 and H3K36me3 by randomly combining fragments from each cancer plasma sample with fragments from pooled healthy-control plasma. Cancer-derived fragments constituted 100%, 50%, 25%, 12.5%, 6.25%, 3.125%, or 0% of each mixture, while total fragment counts were held constant within each patient and histone mark at the limiting depth available after combining the cancer and healthy-control fragment pools. These totals were 43.0 million H3K36me3 and 13 million H3K4me3 fragments for M2246 and 7.4 million H3K36me3 and 1.4 million H3K4me3 fragments for M2180. Three independently sampled replicates were generated at each dilution level using a fixed random seed. APEX features and inferred gene expression were recalculated separately for each dilution replicate.

For each dilution, inferred expression of the 100 signature genes was normalized by the mean inferred expression of a predefined white blood cell gene set. Signature recovery was quantified using the paired t-statistic comparing normalized signature-gene expression at each dilution with that in the 0% cancer-plasma control. Nominal dilution fractions were scaled by the estimated tumor fraction of the undiluted sample (77.4% for M2246 and 21.1% for M2180) to estimate the effective tumor fraction at each dilution. Patient specificity was assessed by comparing recovery of the tumor signature matched to each plasma sample with recovery of the other patient’s unmatched signature. A specificity score was calculated as the matched-signature t-statistic minus the unmatched-signature t-statistic, with positive values indicating preferential retention of the transcriptional program corresponding to the matched tumor biopsy.

### Benchmarking APEX–H3K4me3 against promoter coverage

To ask whether APEX applied to a single histone mark (APEX–H3K4me3) captures lineage-specific transcriptional information beyond raw H3K4me3 promoter coverage, we analyzed plasma cfChIP-seq data from a previously published study^17^. From this dataset, we selected 132 cfChIP-seq samples across colorectal cancer (*n* = 32), ER− breast cancer (*n* = 8), ER+ breast cancer (*n* = 6), hepatocellular carcinoma (*n* = 10), lung adenocarcinoma (*n* = 25), lung squamous cell carcinoma (*n* = 5), neuroendocrine prostate cancer (*n* = 9), and prostate adenocarcinoma (*n* = 37), applying the same strict criteria used in the original study, including H3K4me3 enrichment score >10, >2 million uniquely aligned fragments, and estimated tumor DNA fraction >0.03. We also removed patient-level duplicates and only included cancer subtypes with >3 biological replicates.

Cancer-subtype-specific marker genes were defined using bulk tumor RNA-seq profiles from the corresponding TCGA tumor types by computing a specificity score for each gene as described above (“Defining cancer- and normal-subtype-specific gene signatures”), retaining the top ten genes per cancer subtype. The union of these genes constituted the reference signature set. Plasma-derived APEX–H3K4me3 predicted expression values and raw H3K4me3 promoter coverage values (±3 kb around the TSS, normalized by DHS accessibility^17,77^) were subsetted to the intersecting marker set. For each cancer subtype, discriminatory performance was quantified by comparing the distribution of marker-gene signal in plasma samples from the cancer of interest versus all other cancers using a two-sided Wilcoxon rank-sum test, with −log_10_ (P) values. To visualize lineage-specific programs, we constructed marker-gene-by-cancer-subtype matrices in which each entry corresponds to the plasma signal of an individual signature gene (*n* = 10 per cancer subtype) evaluated across cancer subtypes. For visualization only, matrices were *z*-score normalized and values were smoothed using a centered rolling mean (window size = 3) along the cancer-subtype axis using the R zoo package (v1.8–15).

### Classification of pancreatic basal and classical transcriptional states using APEX

To ask whether APEX-inferred gene expression more accurately discriminates basal versus classical transcriptional subtypes of pancreatic ductal adenocarcinoma (PDAC) than single-mark promoter or gene-body coverage, we applied PurIST, an established logistic-regression classifier trained on bulk tumor RNA-seq^29^. PurIST assigns PDAC samples to basal or classical subtypes using a predefined set of top-scoring gene pairs (TSPs) and is publicly available (https://github.com/naimurashid/PurIST; v02_12_2019^29^). We implemented the classifier using the published R code and released model object, applying the apply_classifier function with default settings to matched matrices of (i) bulk RNA-seq (reference), (ii) APEX-inferred gene expression, (iii) H3K4me3 promoter coverage, and (iv) H3K36me3 gene-body coverage.

Subtype concordance between plasma-derived predictions and tumor-derived PurIST labels was assessed using two-sided Fisher’s exact tests and balanced accuracy. To quantify separation between predicted basal and classical states, PurIST-derived basal probabilities from each modality were compared using two-sided Wilcoxon rank-sum tests.

### SCLC cell-surface target analysis

Baseline APEX-inferred gene expression profiles from 34 patients with SCLC treated with tarlatamab were previously described by Vasseur et al.^30^. SCLC-A, SCLC-N, and SCLC-P subtype assignments were obtained from the same study and were inferred from APEX profiles using a random forest classifier trained on tumor RNA-seq. Cell-surface targets were curated by combining targets of FDA-approved therapies (see “APEX-based nomination of expression-based cancer targets” below) with candidate cancer-specific cell-surface proteins (caGESPs) from Supplementary Table 9 of the Cancer Surfaceome Atlas study^50^. Targets specific to acute myeloid leukemia were excluded. Hematopoietic genes, defined as genes with higher mean expression in blood- and immune-dominant GTEx tissues (including whole blood, EBV-transformed lymphocytes, and spleen) than in other tissues, were also excluded, resulting in 291 non-hematopoietic cancer cell-surface targets (**Table S8**).

Using this curated target set, subtype enrichment for each gene was calculated as the difference between mean APEX-inferred expression in the indicated subtype and mean expression across the remaining subtypes. The 10 genes with the greatest enrichment were selected independently for each subtype.

The resulting 30 APEX-nominated genes were evaluated in an independent cohort of 271 patients with SCLC from the IMpower133 trial using tumor RNA-seq^31^. The same one-versus-rest enrichment statistic was calculated, and concordance was defined as positive enrichment in the APEX-nominated subtype. Statistical significance was assessed using a null distribution generated from 10,000 permutations of IMpower133 subtype labels, from which an empirical *P* value was calculated.

For analysis of therapeutically relevant SCLC targets, APEX-inferred expression of CD276, DLL3, SEZ6, and TACSTD2 was compared with tumor RNA-seq expression from IMpower133. Expression was standardized independently within each cohort, and differences were assessed using two-sided Wilcoxon rank-sum tests.

### Longitudinal analysis of SCLC surface-target expression

Longitudinal analysis was performed in 20 patients with SCLC treated with tarlatamab who had matched plasma samples at baseline (C1D1), early on-treatment (C1D15), and a later on-treatment timepoint. APEX profiles and treatment timepoint definitions were obtained from the previously described cohort of 34 patients reported by Vasseur et al.^30^. The late on-treatment timepoint was defined as the last available sample for each patient and corresponded to either C2D15 or C3D1. At each timepoint, DLL3, SEZ6, and CD276 were evaluated relative to the 95th percentile of their respective distributions in healthy controls. Each sample was assigned the dominant target with the highest APEX-inferred expression among targets exceeding this threshold; samples in which no target exceeded the threshold were classified as having no dominant target. Transitions in dominant target assignment were visualized using Sankey plots generated with ggalluvial (v0.12.6).

### Immunohistochemistry and pathologic scoring

Tumor samples selected for DLL3 and TROP2 immunohistochemistry (IHC) were chosen based on plasma-inferred expression levels derived from APEX, with cases dichotomized into high and low groups using a median cutoff and representative samples taken from the highest and lowest inferred expression values. Sample selection was otherwise restricted only by the availability of matched tumor tissue and the suitability of formalin-fixed paraffin-embedded (FFPE) material for immunohistochemical staining. Pathologic evaluation was performed blinded to plasma-derived expression estimates.

IHC was performed on 3-μm–thick deparaffinized FFPE tissue sections using an automated staining platform (Ventana Benchmark; Ventana Medical Systems, Tucson, AZ, USA) at the PETRA platform, Gustave Roussy. Serial sections were stained with antibodies against DLL3 (clone SP347, prediluted; Roche, France; 08416931001) and TROP2 (clone EPR20043, 1:500; Abcam, USA; ab214488). Stained slides were digitized at ×20 magnification using a Slideview VS200 scanner (Olympus, Tokyo, Japan), ensuring high spatial resolution for evaluation.

DLL3- and TROP2-stained slides were evaluated by a pathologist using standard light microscopy. Cases with major tissue artifacts in the tumor area that precluded reliable evaluation were excluded from analysis. Scoring was performed at ×20 magnification in malignant cells using a semi-quantitative approach incorporating both the percentage of positive tumor cells (0–100%) and staining intensity (0, none; 1+, weak; 2+, moderate; 3+, strong). An H-score was calculated as

H-score=[1×(%cells1+)+2×(%cells2+)+3×(%cells3+)],

yielding a score range from 0 to 300. Any immunoreactivity below 1+ intensity was considered background or non-specific (score 0). DLL3 and TROP2 expression was considered positive when ≥1% of tumor cells demonstrated cytoplasmic and/or membranous staining at any intensity.

Separately, B7-H3 (CD276) protein expression was evaluated by IHC in SCLC patient-derived xenograft (PDX) models representing ASCL1-, NEUROD1-, and POU2F3-defined transcriptional states. PDX models were obtained from the Oser laboratory and generated as previously described^80^. Briefly, patient tumor samples were implanted into 7- to 8-week-old female NSG (NOD.*Cg*-*Prkdc*^*scid*^ *Il2rg*^*tm1Wjl*^/SzJ) mice (The Jackson Laboratory), and established PDX tumors were subsequently expanded and serially passaged subcutaneously in NSG mice. Tumor tissues were formalin-fixed and paraffin-embedded, and 5-μm sections were prepared by the Dana-Farber Cancer Institute Pathology Core. IHC was performed on an automated Leica BOND RX platform using heat-induced epitope retrieval with either citrate buffer (pH 6.0) or EDTA buffer (pH 9.0), as appropriate for each antibody. Primary antibodies were applied for 30 min at ambient temperature, and staining was visualized using polymer-based detection with 3,3′-diaminobenzidine and hematoxylin counterstain. Sections were stained for membranous B7-H3 using a rabbit monoclonal antibody (clone D9M2L, Cell Signaling Technology, catalog no. 14058; 1:100) and for nuclear ASCL1 (clone EPR19840, Abcam, ab211327; 1:100), NEUROD1 (clone EPR20766, Abcam, ab213725; 1:100), and POU2F3 (clone HPA019652, Atlas Antibodies, HPA019652; 1:200). H-scores were calculated as described above.

### APEX-based nomination of expression-based cancer targets

Expression-based therapeutic targets were curated from the U.S. Food and Drug Administration (FDA) website and from a published compendium of cancer cell-surface protein targets^50^. FDA-approved targets were restricted to indications in solid tumors to minimize prioritization of hematopoietic markers arising from background blood signal. *CLDN18* was excluded because the approved antibody zolbetuximab specifically targets the *CLDN18.2* isoform, which cannot be resolved by our existing approach^81^.

The pan-solid cancer cohort comprised all plasma samples included in this study from the MOSCATO–1 and MOSCATO–2 clinical trial cohorts, the pancreatic cancer cohort, the metastatic bladder cancer cohort, the small-cell lung cancer cohort, and the previously published cfChIP-seq cohort^17^. Only one sample per patient was included, selected at random among replicates, with no minimum tumor fraction threshold applied. Samples from the Baca *et al., Nat. Med*., 2023 cohort^17^ were included if they met a permissive quality criterion of enrichment score >7. In total, the analysis included 451 patients with cancer.

Cancer types were summarized into unified categories as follows: Breast (breast cancer); Colorectal (colorectal cancer); Upper gastrointestinal (esophageal adenocarcinoma, esophageal squamous cell carcinoma, small intestine adenocarcinoma, and gastric cancer); Pancreatobiliary (pancreatic cancer, cholangiocarcinoma); Gynecologic (endometrial and ovarian cancer); Liver (hepatocellular carcinoma); NSCLC (lung adenocarcinoma, lung squamous cell carcinoma); SCLC / Neuroendocrine (small-cell lung cancer, neuroendocrine lung tumors, small-cell bladder cancer, Merkel cell carcinoma); Prostate / NEPC (castration-sensitive, castration-resistant, and neuroendocrine prostate cancer); Urothelial (urothelial carcinoma); Central nervous system (glioma); and Other (vesicular adenocarcinoma, melanoma, renal cell carcinoma, mesothelioma, myoepithelial carcinoma, medullary thyroid cancer, head and neck cancer, otherwise unspecified).

APEX inference was performed using H3K4me3 alone when H3K36me3 was unavailable, and using both marks when available, with default model parameters. For each gene, high expression was defined as either the 90^th^ percentile of APEX-inferred expression across the cancer cohort.

### Differential expression and survival analyses in metastatic bladder cancer cohort

Differential expression analysis of APEX-inferred gene expression profiles was performed using the limma R package (v3.62.2)^82^. Gene-level expression estimates were modeled using linear models with group-specific coefficients, enabling direct estimation of expression differences between predefined comparison groups (for example, responders versus non-responders to enfortumab vedotin; **Table S14**). Empirical Bayes moderation was applied to stabilize variance estimates, and log_2_ fold changes and associated *P*-values were computed for all genes.

Survival analyses were performed using the survival R package (v3.8.3)^83^. For univariate analyses, patients were stratified into high and low groups based on median dichotomization of the feature of interest. Progression-free survival and overall survival were evaluated using Kaplan-Meier estimates and compared between groups using two-sided log-rank tests. Hazard ratios (HRs) and 95% confidence intervals (CIs) were estimated using Cox proportional hazards models.

Multivariable Cox proportional hazards models were used to assess whether plasma-inferred molecular features were independently associated with survival outcomes after adjustment for relevant clinical covariates. Candidate covariates were initially evaluated in univariate Cox models, and those showing the strongest univariate associations were prioritized to limit model complexity. The primary multivariable model included APEX-inferred *NECTIN4* expression, presence of visceral metastases (liver and/or brain), and plasma tumor fraction estimated using ichorCNA v0.2.0. Separate multivariable models additionally adjusted for either line of therapy or Karnofsky performance status to determine whether the association between APEX-inferred NECTIN4 expression and survival remained significant after accounting for these clinically relevant factors. Statistical significance was assessed using Wald tests, and forest plots were generated using the forestmodel R package (v0.6.2). All statistical tests were two-sided.

### *NECTIN4* copy-number analysis

*NECTIN4* copy number in the metastatic bladder cancer cohort was inferred from cfDNA sequencing data using ichorCNA v0.2.0^24^. Default parameters were used, except that 500-kb bins were selected to resolve focal copy-number alterations encompassing *NECTIN4*. Copy-number status at the *NECTIN4* locus was obtained from the ichorCNA segment-level output for chr1:161.0–161.5 Mb (hg19), and amplification status was assigned according to the ichorCNA call. *NECTIN4* copy number was compared according to response to enfortumab vedotin, correlated with APEX-inferred *NECTIN4* expression, and evaluated in progression-free and overall survival analyses. A multivariable Cox proportional hazards model including continuous APEX-inferred *NECTIN4* expression and ichorCNA-defined *NECTIN4* amplification status was also fitted.

## STATISTICAL ANALYSIS

No statistical methods were used to predetermine sample size, and cohort sizes were determined by the availability of matched plasma and tumor material. Measurements were obtained from independent biological samples (one patient per sample) unless otherwise indicated. The exact value of *n* and its definition (e.g., number of patients or biological replicates) are specified in the corresponding figure legends. Plasma-based analyses for clinical cohorts were performed blinded to matched tumor reference data and clinical outcomes, and laboratory processing and downstream computational analyses were conducted independently. All statistical tests were two-sided unless otherwise noted, and a *P* value < 0.05 was considered statistically significant. Associations between plasma-derived features and tumor RNA-seq were assessed using Spearman rank correlation; group comparisons were performed using Wilcoxon rank-sum tests, Student’s *t*-tests, or Fisher’s exact tests as appropriate; differential expression analyses were conducted using limma (v3.62.2) with empirical Bayes moderation; and survival outcomes were evaluated using Kaplan–Meier analysis with log-rank testing and Cox proportional hazards models. Machine-learning models were evaluated using cross-validation and independent validation cohorts, with performance assessed by Spearman correlation, root mean squared error, or balanced accuracy as appropriate. Measures of central tendency and dispersion (mean ± SD, median with interquartile range, or 95% confidence intervals) are indicated in the corresponding figures or legends. All analyses were performed in R (v4.4.2) or GraphPad Prism (v10.4.1) using publicly available software with versions specified in the Methods, and routine data manipulation and visualization were performed using ggplot2 (v4.0.1), data.table (v1.17.8), and dplyr (v1.1.4).

## ADDITIONAL RESOURCES

- APEX R package source code, trained models, documentation, and example workflows for gene expression inference from cfChIP-seq data: https://github.com/Baca-Lab/APEX.
- Molecular Screening for Cancer Treatment Optimization (MOSCATO) clinical trial registration (NCT01566019): https://clinicaltrials.gov/ct2/show/NCT01566019.

## Supplementary Material

This is a list of supplementary files associated with this preprint. Click to download.

- APEXsupplement.pdf

Supplementary Tables

Table S1. Comparison of methods for inferring gene expression from ChIP-seq and cell-free epigenomics.

Table S2. Clinical and technical metadata for matched plasma-tumor pairs in the training cohort (MOSCATO–1).

Table S3. Antibodies used for cfChIP-seq, cfMeDIP-seq, and immunohistochemistry.

Table S4. Genomic coordinates of regulatory regions used in this study.

Table S5. Results of an in silico screen of cfChIP-seq chromatin features associated with tumor gene expression.

Table S6. Ranked feature importance in the APEX model based on random permutation analysis.

Table S7. Clinical and technical metadata for matched plasma-tumor pairs in the test cohort (MOSCATO–2).

Table S8. Gene signatures for cancer- and tissue-specific analyses.

Table S9. Sample-level metadata and gene-level expression estimates used for benchmarking plasma-based inference methods.

Table S10. Clinical and technical metadata for samples from a previously published pan-cancer plasma epigenomics study.

Table S11. Clinical, technical, and subtype classification metadata for matched plasma-tumor pairs in the metastatic pancreatic cancer cohort.

Table S12. Clinical and technical metadata for longitudinal plasma cohort of SCLC patients treated with tarlatamab.

Table S13. Clinical and technical metadata for baseline plasma cohort of metastatic bladder cancer patients treated with enfortumab vedotin.

Table S14. APEX differential expression analysis between responders and non-responders to enfortumab vedotin in metastatic bladder cancer.

Table S15. Sequencing and alignment quality-control metrics for all analyzed samples.

Supplementary Data

Data S1. APEX-inferred gene expression profiles for the MOSCATO–1 training cohort.

Data S2. Bulk tumor RNA-seq gene expression profiles (logTPM) for the MOSCATO–1 training cohort.

Data S3. APEX-inferred gene expression profiles for the MOSCATO–2 test cohort.

Data S4. Bulk tumor RNA-seq gene expression profiles (logTPM) for the MOSCATO–2 test cohort.

Data S5. APEX-inferred gene expression profiles for limit-of-detection dilution series experiments.

Data S6. APEX-inferred gene expression profiles for the metastatic pancreatic cancer cohort.

Data S7. Bulk tumor RNA-seq gene expression profiles (logTPM) for the metastatic pancreatic cancer cohort.

Data S8. APEX-inferred gene expression profiles for the cohort of metastatic small-cell lung cancer patients treated with tarlatamab.

Data S9. APEX-inferred gene expression profiles for the cohort of metastatic bladder cancer patients treated with enfortumab vedotin.

Data S10. APEX-inferred gene expression profiles for samples from a previously published pan-cancer plasma epigenomics study.

Data S11. APEX-inferred gene expression profiles from healthy volunteers.

## Figures and Tables

**Figure 1.**
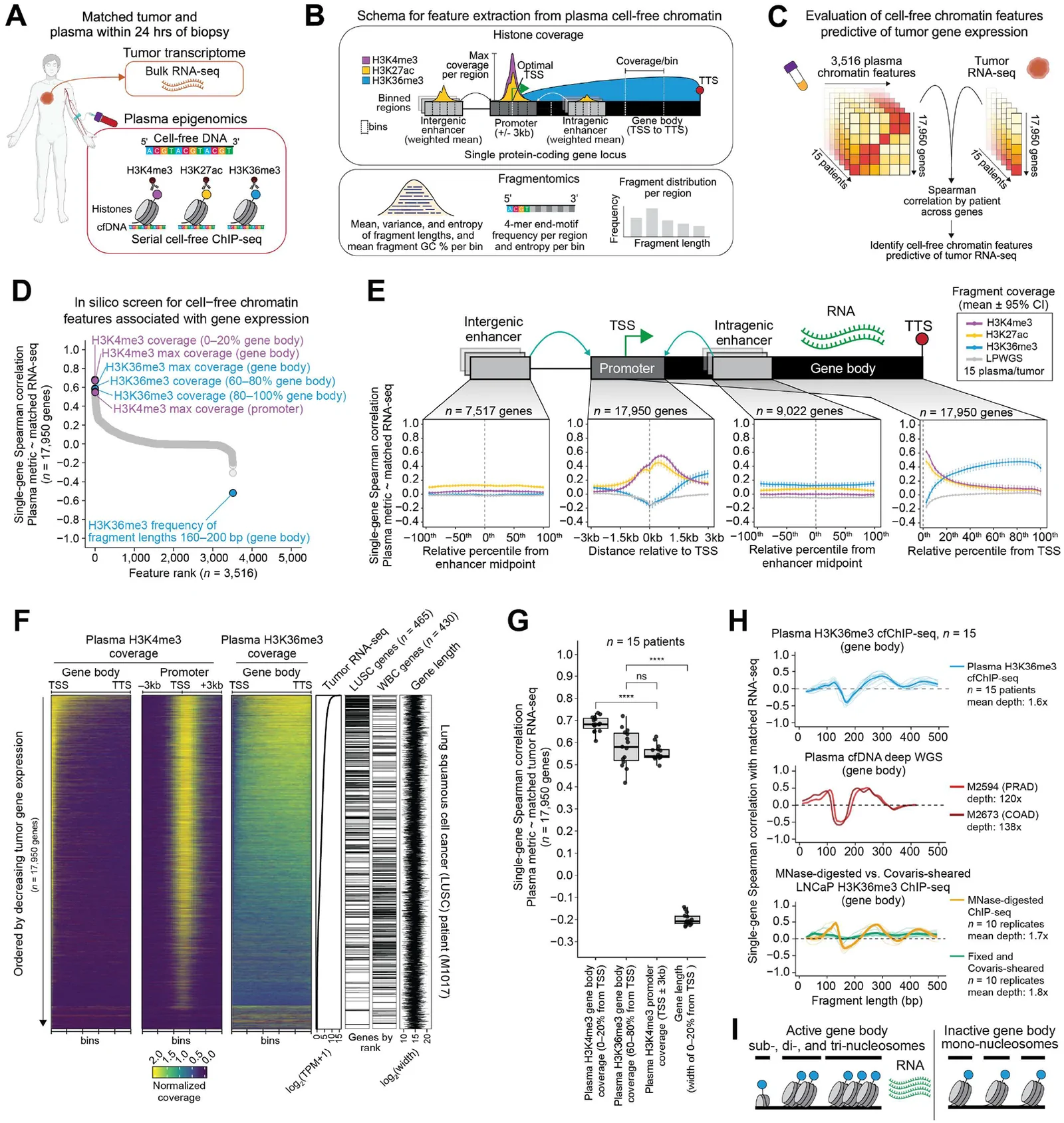
Plasma cell-free chromatin features reflect tumor gene expression. **(A)** Study design for 15 patients with matched tumor biopsies and plasma collected within 24 hours. Tumors were profiled by bulk RNA-seq and plasma by low-pass whole-genome sequencing (LPWGS) and serial cell-free ChIP-seq for H3K4me3, H3K27ac, and H3K36me3. **(B)** Schema for extraction of 3,516 plasma chromatin and fragmentomic features across 17,950 protein-coding genes, including histone coverage across promoters, gene bodies, and enhancers, and regional fragment-length, end-motif, and GC-content features. The optimal transcription start site (TSS) was selected based on H3K4me3 coverage. TTS, transcription termination site. **(C)** Strategy for identifying plasma cell-free chromatin features associated with matched tumor gene expression by Spearman correlation across genes and patients. **(D)** Systematic screen of 3,516 plasma cell-free chromatin features ranked by their correlation with matched tumor RNA-seq across 17,950 genes. Selected highly associated features are highlighted. **(E)** Single-gene correlations between plasma H3K4me3, H3K27ac, H3K36me3, or LPWGS coverage and matched tumor RNA-seq across enhancers, promoters, and gene bodies. Points show mean correlations across 15 patients; error bars indicate 95% confidence intervals. **(F)** Plasma H3K4me3 and H3K36me3 coverage across 17,950 genes in a representative patient with lung squamous cell carcinoma, ordered by decreasing matched tumor RNA-seq expression. Tumor expression, ranks of LUSC- and white-blood-cell-associated genes, and gene length are shown alongside. **(G)** Single-gene correlations between matched tumor RNA-seq and selected plasma chromatin features or corresponding genomic length across 15 patients. Statistical significance was assessed by two-sided paired Wilcoxon tests (****P < 0.0001; ns, not significant). **(H)** Relationship between fragment length and correlation with gene expression for plasma H3K36me3 cfChIP-seq (top), plasma deep WGS (middle), and MNase-digested or Covaris-sheared LNCaP chromatin (bottom). Lines represent individual samples or replicates; bold lines indicate means where shown. **(I)** Model illustrating how transcription-associated nucleosome organization gives rise to fragment-length patterns that distinguish active from inactive gene bodies.

**Figure 2.**
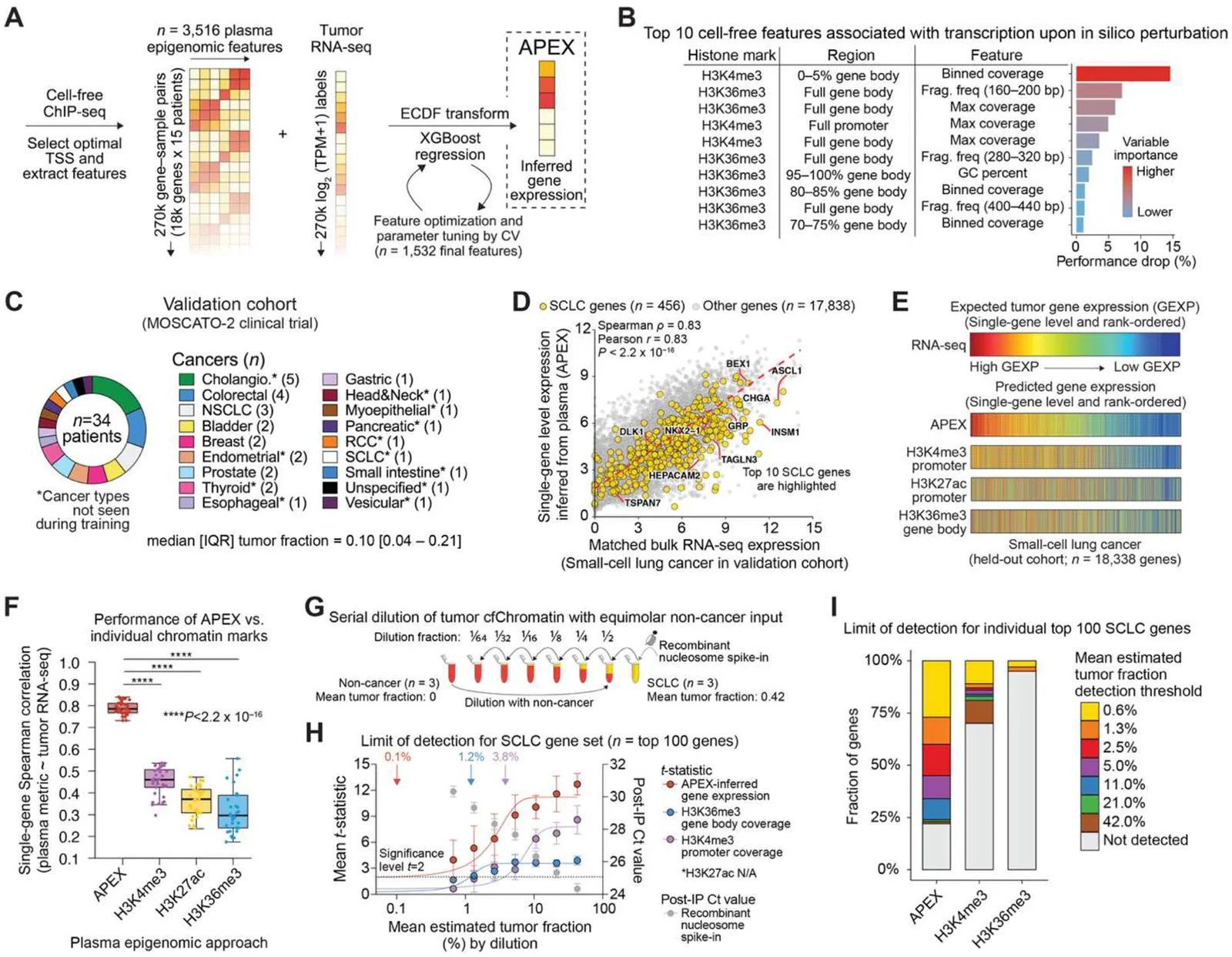
Development, validation, and analytical sensitivity of APEX. **(A)** Overview of the APEX model for inferring tumor gene expression from plasma cell-free chromatin features. **(B)** Top 10 plasma cell-free chromatin features ranked by permutation importance, measured as the decrease in model performance following feature perturbation. **(C)** Independent validation cohort comprising 34 patients across multiple cancer types. Asterisks indicate cancer types not represented in the training cohort. **(D)** APEX-inferred versus matched tumor RNA-seq expression across 18,338 genes in a representative SCLC sample. SCLC-associated genes are highlighted and the 10 most specific genes labeled. Spearman and Pearson correlations are shown. **(E)** Rank-ordered tumor RNA-seq, APEX-inferred expression, and individual plasma histone features for the SCLC sample in (**D**). **(F)** Single-gene correlations with matched tumor RNA-seq for APEX and individual plasma histone features across the 34-patient validation cohort. Statistical significance was assessed by two-sided paired Wilcoxon tests (****P < 0.0001). **(G)** Experimental design for analytical sensitivity testing by serial dilution of cell-free chromatin from three patients with SCLC into cell-free chromatin from three non-cancer individuals. Recombinant nucleosomes served as dilution controls. **(H)** Detection of a 100-gene SCLC signature across serial dilutions using APEX and individual histone features. Points show mean ± s.e.m. across biological replicates; curves indicate sigmoid fits. The dashed line indicates the prespecified detection threshold (*t* = 2), with estimated limits of detection shown above. **(I)** Limits of detection for 100 individual SCLC genes using APEX, H3K4me3, or H3K36me3. Colors indicate the lowest estimated tumor fraction at which each gene was detected; gray indicates genes not detected.

**Figure 3.**
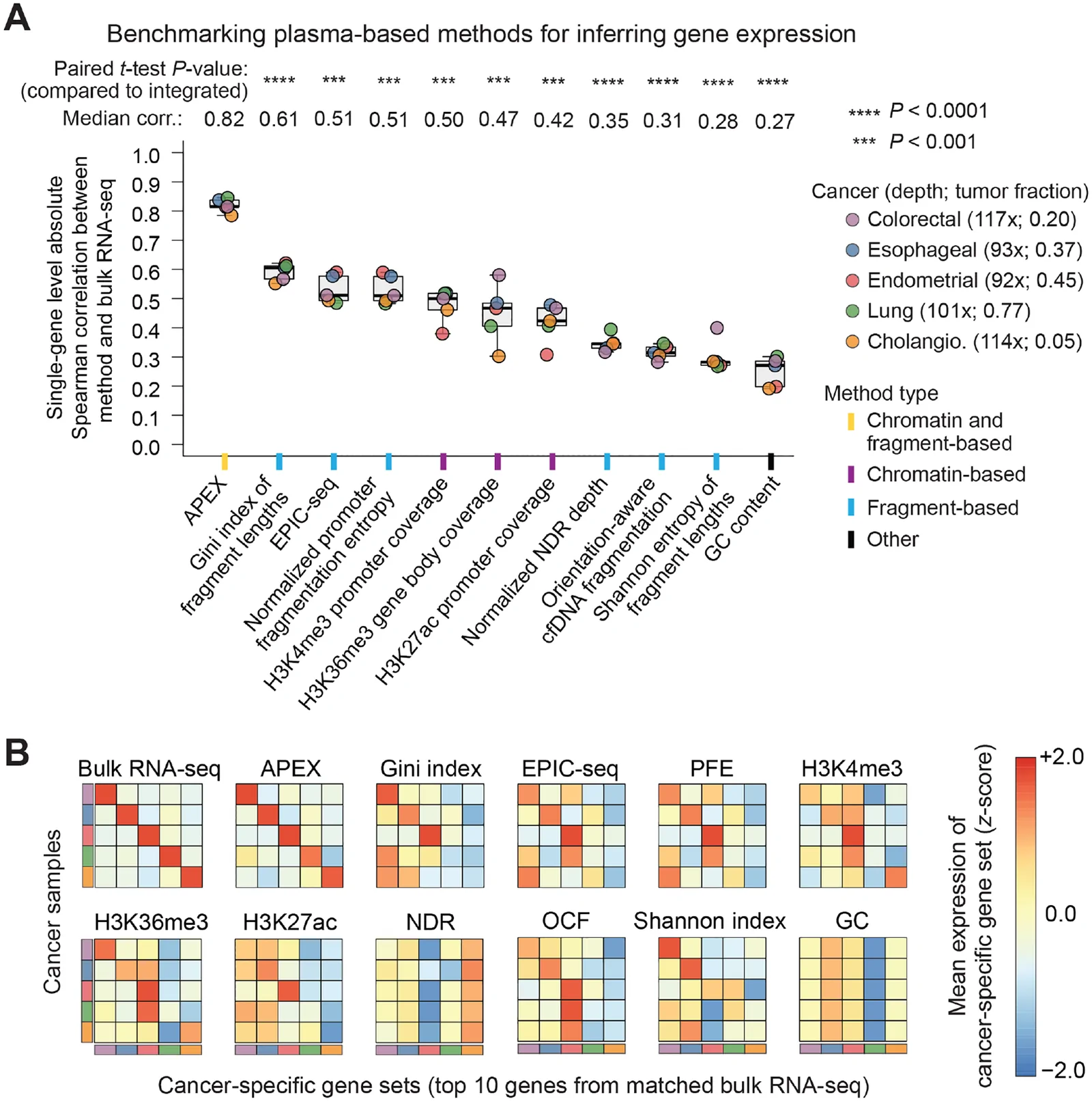
Benchmarking APEX against plasma-based approaches for tumor gene-expression inference. **(A)** Comparison of APEX with chromatin- and fragmentomics-based approaches using matched plasma and tumor RNA-seq from five patients profiled by cfChIP-seq and deep whole-genome sequencing. Performance was assessed by single-gene Spearman correlation across 18,388 genes. Points represent patients; box plots show median, quartiles, and 1.5× IQR. Median correlations and paired *t*-test *P* values versus APEX are shown above. **(B)** Recovery of cancer-specific gene-expression patterns by each plasma-based approach. For each cancer, the top 10 cancer-specific genes were defined from matched tumor RNA-seq and their inferred expression was z-score normalized across samples. NDR, nucleosome-depleted region; OCF, orientation-aware cfDNA fragmentation.

**Figure 4.**
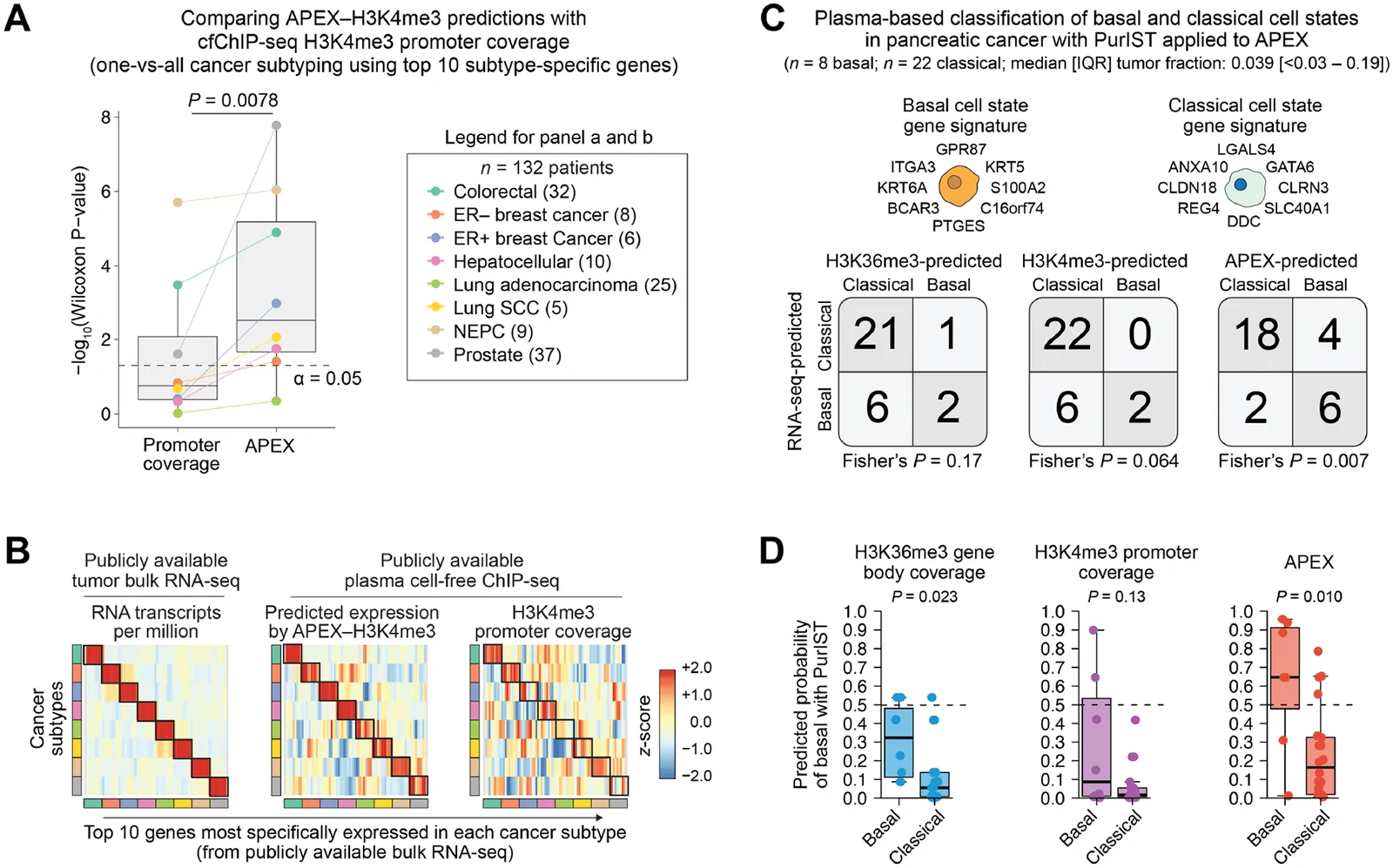
APEX discriminates cancer subtypes and transcriptional cell states from plasma. **(A)** Comparison of APEX–H3K4me3 with plasma H3K4me3 promoter coverage for distinguishing eight cancer subtypes across 132 patients^17^ using the top 10 subtype-specific genes defined from public tumor RNA-seq^48^. Points indicate cancer subtypes; paired significance was assessed by two-sided Wilcoxon test. Dashed line indicates *P* = 0.05. **(B)** Heatmaps showing subtype-specific gene expression in public tumor RNA-seq (left)^48^, APEX–H3K4me3-inferred expression (center), and plasma H3K4me3 promoter coverage (right). The top 10 genes for each subtype were defined from tumor RNA-seq. **(C)** Classification of basal versus classical pancreatic cancer states using PurIST^29^ applied to plasma H3K36me3 gene-body coverage, H3K4me3 promoter coverage, or APEX-inferred expression, compared with matched tumor RNA-seq (n = 30). *P* values are from Fisher’s exact tests. **(D)** PurIST-predicted probability of the basal state for the same patients in (C). Dashed line indicates the 0.5 classification threshold. *P* values are from two-sided, unpaired *t*-tests. Box plots in (**A**) and (**D**) show medians, quartiles, and 1.5 × IQR.

**Figure 5.**
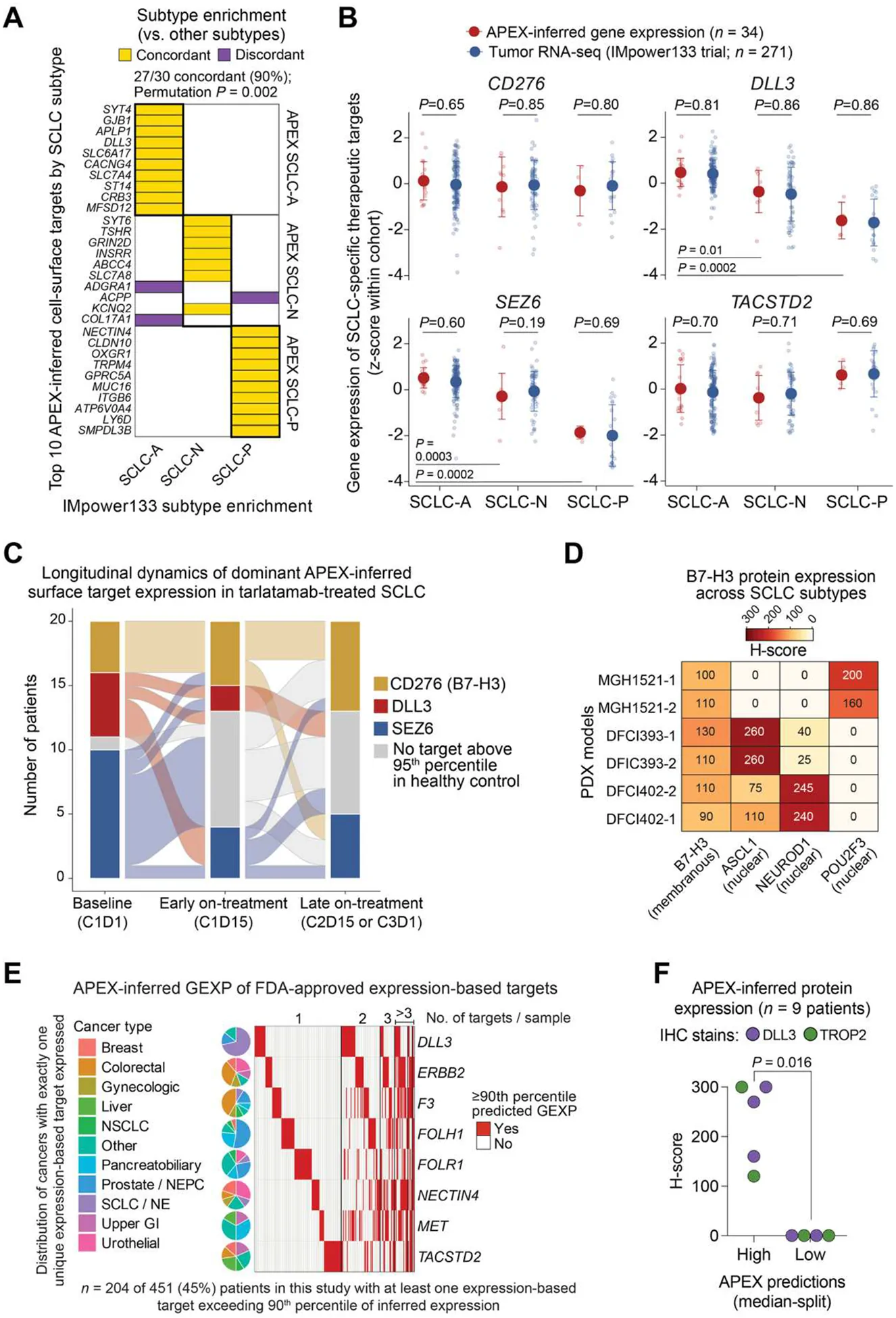
APEX resolves therapeutically relevant cell-surface-target heterogeneity and dynamics in SCLC and across cancer types. **(A)** Top 10 APEX-inferred cell-surface genes enriched in each SCLC subtype using baseline plasma samples from 34 patients prior to tarlatamab treatment, and validation of their same-subtype enrichment by tumor RNA-seq from the IMpower133 trial (n = 271). Yellow indicates concordant and purple discordant subtype enrichment. An empirical P value was calculated from a null distribution generated by 10,000 permutations of the IMpower133 subtype labels. **(B)** APEX-inferred expression of CD276 (B7-H3), DLL3, SEZ6, and TACSTD2 (TROP2) across SCLC subtypes (*n* = 34) compared with tumor RNA-seq from IMpower133 (*n* = 271). Expression was standardized within each cohort. Points show individual samples and mean ± s.d. *P* values are from two-sided Wilcoxon rank-sum tests; values above groups compare APEX with RNA-seq, with selected subtype comparisons shown below. **(C)** Sankey plot showing longitudinal dynamics of APEX-inferred dominant surface targets during tarlatamab treatment. Ribbons track individual patients from baseline (C1D1) through early (C1D15) and late (C2D15 or C3D1) treatment. At each timepoint, the dominant target was defined as the highest-expressed of DLL3, SEZ6, and CD276 that exceeded the 95th percentile in healthy controls; samples without a target exceeding this threshold were classified as no target. **(D)** B7-H3 protein expression by IHC across SCLC patient-derived xenograft models representing distinct transcriptional subtypes. Values indicate H-scores. **(E)** Heatmap showing APEX-inferred gene expression (GEXP) of targets of FDA-approved expression-based therapies across 451 plasma samples. Red indicates samples with inferred expression at or above the 90th percentile for each target. Samples are ordered by the number of elevated targets (top); pie charts show the cancer-type distribution among samples with exactly one elevated target. **(F)** IHC validation of APEX predictions. DLL3 and TROP2 H-scores from nine tumors are stratified by high versus low APEX-inferred expression using a median split. *P* value was calculated by two-sided Wilcoxon rank-sum test.

**Figure 6.**
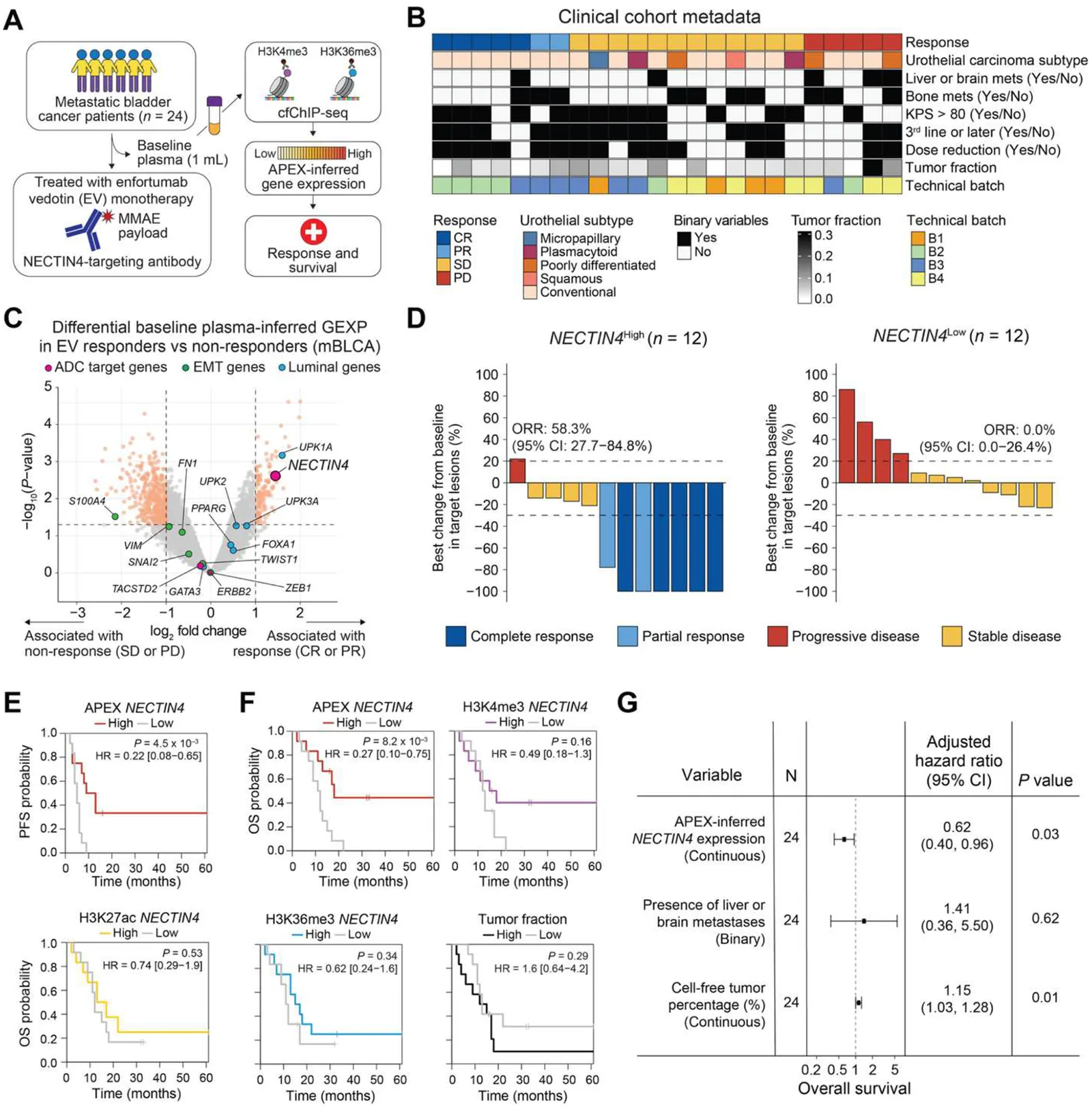
APEX-inferred NECTIN4 expression predicts response and survival to enfortumab vedotin. **(A)** Study design for 24 patients with metastatic bladder cancer treated with enfortumab vedotin (EV) monotherapy, with baseline plasma profiled by cfChIP-seq and APEX. **(B)** Clinical and pathological characteristics of the EV-treated cohort. **(C)** Differential baseline APEX-inferred gene expression between responders (CR or PR) and non-responders (SD or PD). Selected ADC targets, epithelial–mesenchymal transition genes, and luminal lineage genes are highlighted. **(D)** Best percentage change in target lesions according to high versus low baseline APEX-inferred *NECTIN4* expression (median split). Objective response rates (ORR) and 95% confidence intervals are shown. **(E)** Progression-free survival according to high versus low APEX-inferred *NECTIN4* expression. **(F)** Overall survival according to APEX-inferred *NECTIN4* expression, individual *NECTIN4* cfChIP-seq features, and plasma tumor fraction. *P* values are from two-sided log-rank tests; hazard ratios (HR) and 95% confidence intervals are shown. **(G)** Multivariable Cox proportional hazards analysis of overall survival including continuous APEX-inferred *NECTIN4* expression, liver or brain metastases, and plasma tumor fraction. Adjusted HRs and 95% confidence intervals are shown.
